# The Economics of a “portion size reduction” policy

**DOI:** 10.1371/journal.pone.0279165

**Published:** 2022-12-15

**Authors:** Hanin Hosni, Konstantinos Giannakas

**Affiliations:** Department of Agricultural Economics, University of Nebraska-Lincoln, Lincoln, Nebraska, United States of America; Universitat Jaume I, SPAIN

## Abstract

This study develops novel models of heterogeneous consumer preferences for different dining options and imperfect competition among food suppliers to analyze the market and welfare effects of portion size reduction (PSR) for food away from home. Different scenarios on the nature of differentiation of the dining options, the information available to consumers, and their response to links between portion size and obesity, food waste, and climate change are considered within this framework. The market and welfare effects of the policy are quantified using a simulation analysis. The analysis shows that the market and welfare effects of the policy are case-specific and dependent on the relative magnitude of the cost and utility effects of PSR, the strength of the consumer preference for dining out, and the food suppliers’ initial costs and degree of market power in the relevant markets. The policy can create winners and losers among consumers and accounting for consumer heterogeneity, as done in this study, is essential for capturing the asymmetric welfare effects of PSR. Intriguingly, consumers and suppliers can benefit from PSR even without accounting for any health or/and environmental benefits of reduced portion sizes.

## 1. Introduction

The portion size of common food items consumed at home, restaurants, and fast-food establishments in the United States (US) has increased since the 1970s [1], with the portion size of meals and beverages in several restaurants exceeding the US Department of Agriculture (USDA) and Food and Drug Administration recommendations [2]. Portion size has continued to grow in parallel with increasing body weights and food waste. According to USDA, 35% of the US population suffers from obesity while 40 million people are food insecure [3]. At the same time, about 1/3 of the US food supply goes unconsumed, with 2/3 of food waste occurring within the household [4] and the remaining 1/3 occurring in retail stores and food services [5]. The restaurant service sector wastes up to 10% of purchased food before it reaches the final consumer [6] and 21% of the food served in restaurants is not being eaten [4]. Food is the single largest component ending up in landfills accounting for 22% of municipal solid waste, which is an important source of greenhouse gases emissions that cause climate change [7].

Previous studies have shown that increased portion sizes lead to both increased food intake–which contributes to overweight/obesity–and increased food waste [8–11]. Additionally, increased portion sizes can distort consumer perceptions about “appropriate” food portions [12]. This ‘portion size effect’ is sustained when people are exposed to larger portions for several days [13] or weeks [14] and has prompted calls for portion size reductions [1, 9, 15–17].

Research suggests that, in the same way that the availability of supersized portions has normalized larger portions [18], reducing food portion sizes might recalibrate people’s perception of normal portion size and induce the selection of smaller portions in the future [19]. Experimental evidence suggests that, following the manipulation of external food environmental factors, including portion size, food intake is poorly adjusted [9]. For instance, in their 5-day laboratory experiment, Haynes and colleagues found that reductions in the portion size of main meals resulted in a significant decrease in daily food intake. Additional food consumption did not offset this reduction even when portions were reduced to the point where they were no longer perceived as normal [8]. Similarly, Rolls et al. (2006) served participants meals that were 25% reduced in portion size across 2 days. Their findings suggest that, even though participants reduced their caloric intake by 231 kcal per day, they reported similar ratings of hunger and fullness over the 2 days [10].

Despite its effectiveness in reducing both food intake and food waste, a systematic analysis of the market and welfare impacts of implementing a policy of “Portion Size Reduction” has, to our knowledge, not been considered in the literature.

This study addresses this issue/gap in the literature and determines the system-wide market and welfare effects of reducing portion sizes in the US foodservice industry. In particular, the objectives of the study are to examine the impacts of portion size reduction (PSR) on the prices and market shares of food away from home (FAFH) and food at home (FAH), as well as the impact of smaller portions on the welfare of the interest groups involved (i.e., consumers and food suppliers).

To analyze the system-wide economic impacts of PSR, the study develops product differentiation models that explicitly account for differences in consumer preferences for the different dining options and imperfect competition in the food industry. Different scenarios on the nature of differentiation of the dining options, the information available to consumers and their response to links between portion size and obesity, food waste, and climate change are considered within this framework.

A reduction in portion sizes can be expected to reduce the consumer valuation of, and, thus, the willingness-to-pay for food prepared away from home (Utility or Demand Effect of PSR, see [20]) as well as the costs faced by the relevant foodservice providers (Cost or Supply Effect of PSR). While the reduced demand and costs (due to reduced portion size) will always reduce the prices of FAFH and FAH, the impact of PSR on the quantities and market shares of the two dining options, food providers’ profits, and consumers’ welfare are shown to depend on the relative magnitude of the demand and supply effects of the PSR. This study identifies all possible scenarios on the market and welfare impacts of reduced portion size as well as the exact conditions under which each scenario will emerge. The study also considers the impact of providing information to consumers about the rationale of PSR, the links between portion size and obesity, food waste, and climate change, and the impact of such information on the market and welfare effects of the policy. Finally, using observed data on prices, expenditure shares and profit margins, we derive market share and cost estimates, calibrate the theoretical PSR model and carry out a simulation analysis to quantify the market and welfare impacts of PSR. Before concluding this part, it is important to note that, while our study focuses on the market and welfare impacts of the introduction of a “portion size reduction” policy in the US, the analytical results are more general and apply to all cases/countries where portion sizes are large/excessive and associated with increased incidence of obesity, food waste and/or greenhouse gas emissions.

The rest of this paper is organized as follows. The next section presents the theoretical framework and the equilibrium conditions for the benchmark case prior to the reduction in portion size. The sections following derive the equilibrium conditions after the introduction of the policy and determine the market and welfare impacts of PSR and the impact of information provision on the market and welfare effects of the policy. The simulation analysis is presented and discussed before the final section summarizes and concludes the paper.

## 2. Framework of analysis

Our model considers two interest groups: food suppliers (i.e., restaurants, grocery stores), and consumers. The two dining options (i.e., FAFH and FAH) are modeled as vertically differentiated, i.e., uniformly quality-ranked by consumers so that, if offered at the same price, all consumers would prefer to have someone else, a professional cook for them. While all consumers prefer having food prepared by a professional, consumers differ in their valuation of the perceived quality difference between the different dining options. After the reduction in the portion size of FAFH options, consumer valuation of these options is expected to change based on the strength of their preference for food prepared away from home. The model is an adaptation of the vertical product differentiation framework presented in [21].

To determine the market and welfare effects of the PSR, we compare prices, quantities/market shares, and interest groups’ welfare before and after the policy introduction accounting for both the demand and supply effects of the policy. As noted earlier, the *demand/utility effect* refers to the change in the consumer valuation of food prepared away from home due to the reduction in the portion sizes, while the *supply/cost effect* of the PSR refers to the cost reduction for the FAFH suppliers due to the decrease in the product size (e.g., smaller burger, smaller bread buns, fewer veggies, smaller packaging etc.).

Before concluding this part, it is important to note that, while our analysis focuses on the case of vertically differentiated FAH and FAFH, the results of our study are more general as they also hold for the case in which the two dining options are horizontally (rather than vertically) differentiated; i.e., non-uniformly utility-ranked by consumers so that, if offered at the same price, they would both enjoy positive shares of the market (see S1 Appendix). The robustness of our results to the nature of differentiation of the two dining options lies in the fact that the market, in both cases, is covered, i.e., consumers will eat either at home or away from home.

### 2.1. Benchmark case: Pre-portion size reduction (pre-PSR)

#### 2.1.1. Consumer behavior

Consider a consumer that has the choice between two dining options, FAFH (e.g., fast food, casual dining, fine dining, etc.) and FAH (i.e., home cooked meals using grocery store supplies). While food deliveries are generally consumed at home, for the purposes of this study they are considered FAFH as the meals are prepared away from home and their portion size is determined by the food suppliers that prepare these meals. Let *α* ∈ [0,1] be the attribute that differentiates consumers with higher values of *α* corresponding to higher consumer valuation of FAFH–i.e., the greater is *α*, the stronger the consumer preference for FAFH. Assuming that consumers spend a small share of their income on a meal, their utility function is given by:

$$\begin{array}{cc} U_{a}=U-P_{a}+\lambda \alpha & \text{if}\;\text{a}\;\text{meal}\;\text{prepared}\;\text{away}\;\text{from}\;\text{home}\;\text{is}\;\text{consumed}\\ U_{h}=U-P_{h} & \text{if}\;\text{a}\;\text{meal}\;\text{prepared}\;\text{at}\;\text{home}\;\text{is}\;\text{consumed} \end{array}$$ (1)

where *U*_*a*_ and *U*_*h*_ are the utilities associated with the consumption of FAFH and FAH, respectively. *U* is the base level of utility associated with a meal consumption, and *P*_*a*_ and *P*_*h*_ are the consumer prices of FAFH and FAH, respectively. The parameter *λ* is a non-negative utility enhancement factor associated with the consumption of FAFH. To save on notation, *U*_*h*_ is assumed constant across consumers. In this context, the terms *U* + *λα* and *U* represent the consumer willingness-to-pay for a unit of FAFH and FAH, respectively. Subtracting the relevant prices from these willingness-to-pay values, we get the consumer welfare associated with the two dining options.

**Fig 1:**
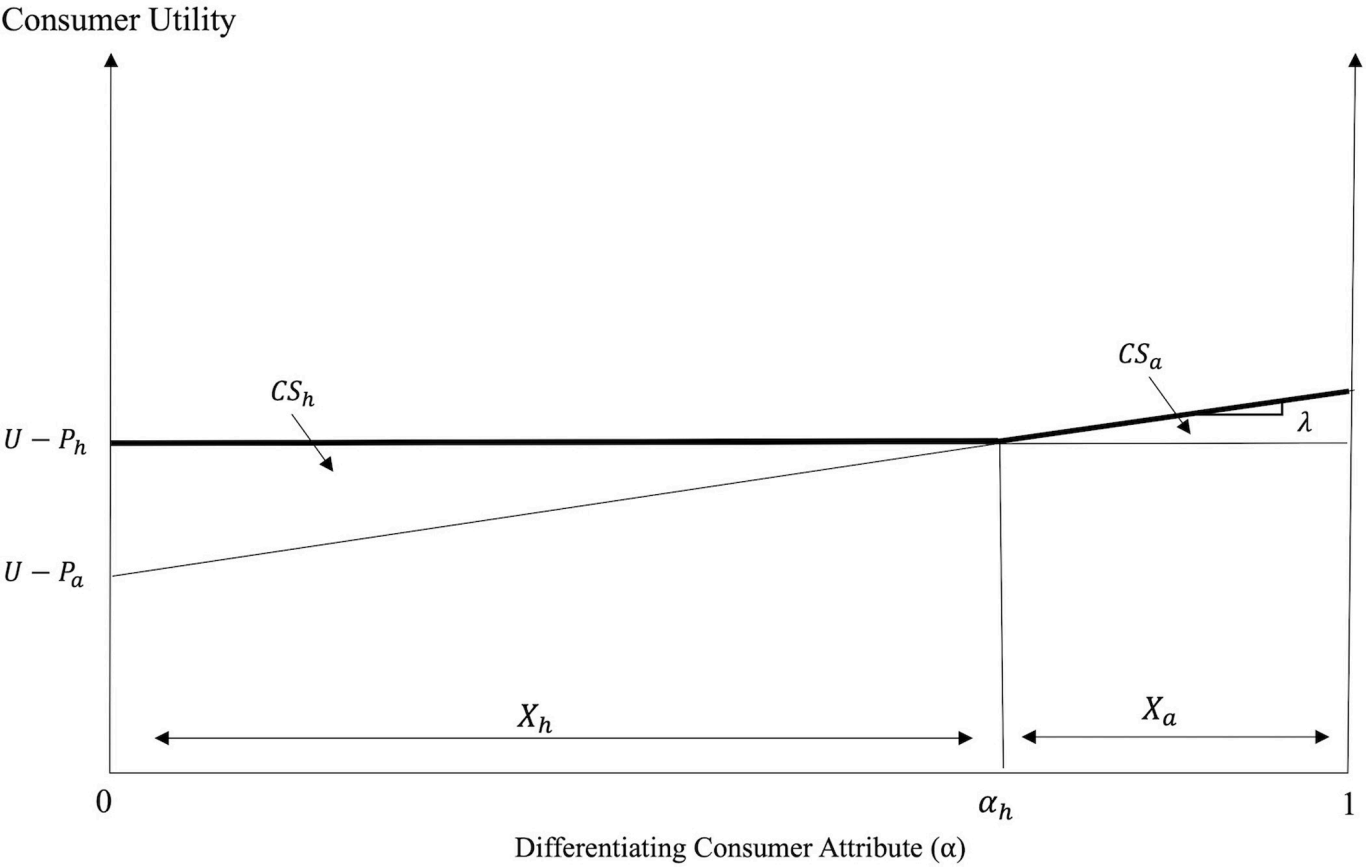
Consumption decisions and welfare before portion size reduction (pre-PSR).

The consumers’ decision depends on the utilities associated with the two dining options. Fig 1 graphs the utilities associated with the consumption of FAH and FAFH meals when the two dining options coexist in the market.

The consumer with differentiating attribute *α*_*h*_: *U*_*h*_ = *U*_*a*_ is indifferent between the two dining options, where

$$\alpha_{h}=\frac{P_{a}-P_{h}}{\lambda }$$ (2)

Consumers with *α* ∈ [0, *α*_*h*_) prefer FAH, while consumers with *α* ∈ (*α*_*h*_,1] prefer FAFH (see Fig 1). When consumers are uniformly distributed with respect to *α* and their mass is normalized to unity, *α*_*h*_ and 1 – *α*_*h*_ give the market shares of, and the consumer demands for the FAH and the FAFH, respectively, as:

$$X_{h}=\frac{P_{a}-P_{h}}{\lambda }$$ (3)

$$X_{a}=\frac{\lambda -P_{a}+P_{h}}{\lambda }$$ (4)

Eqs (3) and (4) show that the demand for FAH (FAFH) decreases with an increase in its own price and/or an increase (decrease) in the strength of the consumer preference for FAFH and increases when the price of the FAFH (FAH) increases. For FAH and FAFH to coexist in the market, the price premium of FAFH must be less than the consumer valuation of the perceived quality difference between the two dining options for all consumers (i.e., $P_{a}-P_{h}<\lambda$).

The area under the bold kinked utility curve in Fig 1 reflects the consumer welfare when the two dining options coexist in the market. The welfare of consumers of FAH and FAFH, *CW*_*h*_ and *CW*_*a*_, respectively, is given by:

$$CW_{h}=\int_{0}^{\alpha_{h}}U_{h}\;d\alpha =\alpha_{h}(U-P_{h})$$ (5)

$$CW_{a}=\int_{\alpha_{h}}^{1}U_{a}\;d\alpha =(U-P_{a}+\frac{\lambda }{2})-\alpha_{h}(U-P_{a}+\frac{\lambda \alpha_{h}}{2})$$ (6)

Aggregate consumer welfare is, then:

$$CW=CW_{h}+CW_{a}=(U-P_{a})+\alpha_{h}(P_{a}-P_{h})+\frac{\lambda }{2}(1-\alpha^{2}{}_{h})$$ (7)

Finally, the surplus of each consumer group is determined by the benefit from its dining choice relative to its alternative as

$$CS_{h}=\int_{0}^{\alpha_{h}}\left(U_{h}-U_{a}\right)\;d\alpha =\alpha_{h}\left(P_{a}-P_{h}-\frac{\lambda \alpha_{h}}{2}\right)$$ (8)

$$CS_{a}=\int_{\alpha_{h}}^{1}\left(U_{a}-U_{h}\right)\;d\alpha =\left(P_{h}-P_{a}+\frac{\lambda }{2}\right)-\alpha_{h}\left(P_{h}-P_{a}+\frac{\lambda \alpha_{h}}{2}\right)$$ (9)

Aggregate consumer surplus is, then, given by:

$$CS=CS_{h}+CS_{a}=\left(P_{a}-P_{h}\right)\left(2\;\alpha_{h}-1\right)+\lambda \left(\frac{1}{2}-\alpha {²}_{h}\right)$$ (10)

#### 2.1.2. Market equilibria

**Fig 2:**
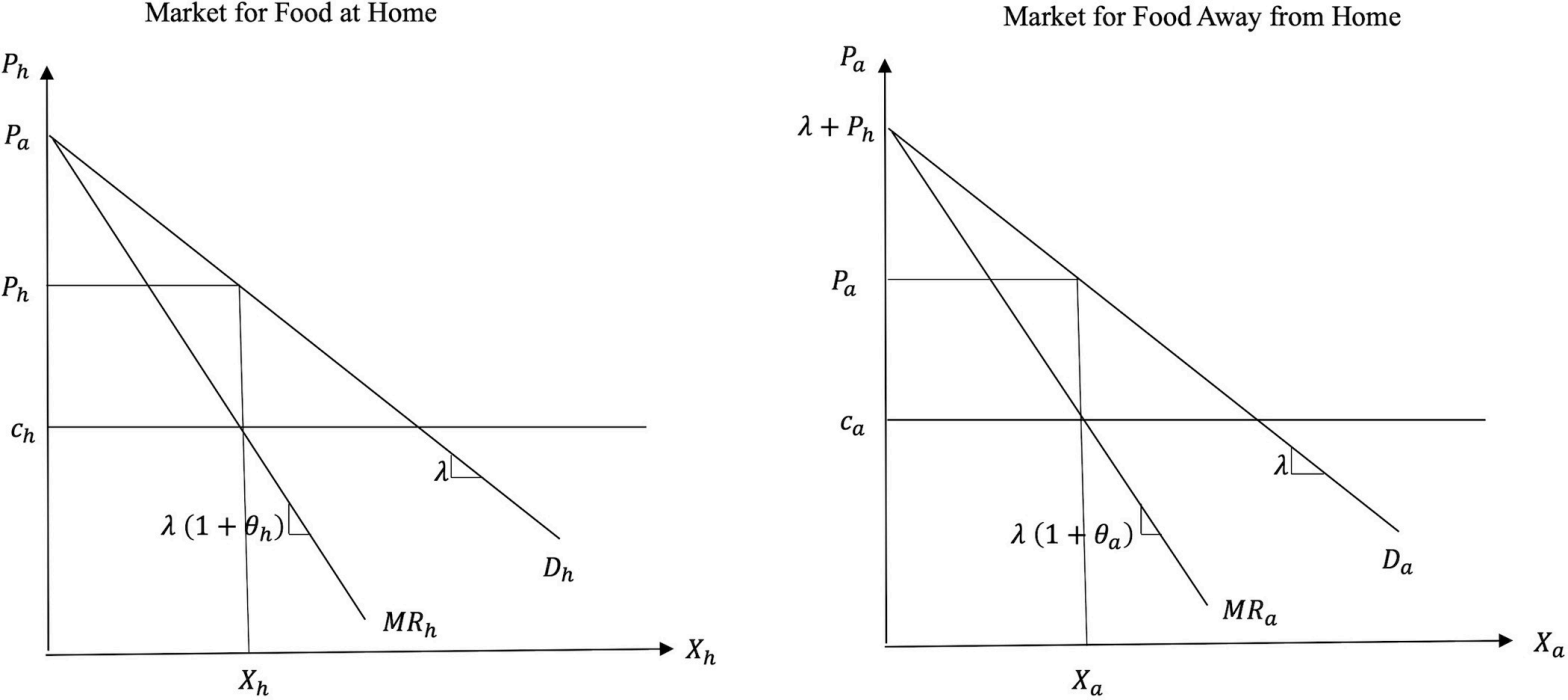
Equilibrium conditions before portion size reduction (pre-PSR).

Fig 2 graphs the inverse demand curves for FAH and FAFH, *D*_*h*_ and *D*_*a*_, respectively, as well as the equilibrium conditions in the two markets in the price-quantity space.

The inverse demand curves for FAH and FAFH are derived from Eqs (3) and (4) and are given by:

$$P_{h}=P_{a}-\lambda \;X_{h}$$ (11)

$$P_{a}=\lambda +P_{h}-\lambda \;X_{a}$$ (12)

Based on the inverse demand functions, the marginal revenue (*MR*) schedules for FAH and FAFH are given by:

$$MR_{h}=P_{a}-\lambda \;\left(1+\theta_{h}\right)\;X_{h}$$ (13)

$$MR_{a}=\lambda +P_{h}-\lambda \;\left(1+\theta_{a}\right)\;X_{a}$$ (14)

where the parameters *θ*_*h*_ and *θ*_*a*_ capture the market power of suppliers of FAH and FAFH, respectively. Parameters *θ*_*h*_ and *θ*_*a*_ are conjectural variation elasticities capturing the degree of market power of the suppliers of FAH and FAFH, respectively, and take values between zero and one. The greater is the value of *θ*, the greater is the market power of the food product suppliers. In particular, values of *θ* *ϵ* (0, 1) capture various oligopolistic market structures, *θ =* 1 captures a monopoly, while *θ =* 0 reflects a perfectly competitive market structure [22].

Equating the marginal revenues with the marginal costs faced by the suppliers of FAH and FAFH (*c*_*h*_ and *c*_*a*_, respectively) we get the equilibrium quantities/market shares as:

$$X_{h}=\frac{P_{a}-c_{h}}{\lambda \;\left(1+\theta_{h}\right)}$$ (15)

$$X_{a}=\frac{\lambda +P_{h}-c_{a}}{\lambda \;\left(1+\theta_{a}\right)}$$ (16)

The costs faced by the FAH and the FAFH suppliers are assumed constant and capture the processing, distribution, and marketing costs in the two supply channels, as well as the market power at earlier stages of the supply chain (e.g., wholesalers).

Substituting *X*_*h*_ and *X*_*a*_ into the inverse demand functions in Eqs (11) and (12), we obtain the prices of the two dining options as:

$$P_{h}=\frac{P_{a}\theta_{h}+c_{h}}{1+\theta_{h}}$$ (17)

$$P_{a}=\frac{\lambda \;\theta_{a}+P_{h}\theta_{a}\;+c_{a}}{1+\theta_{a}}$$ (18)

Solving *P*_*h*_ and *P*_*a*_ simultaneously, we derive the equilibrium prices of FAH and FAFH as a function of the exogenous parameters of our model (i.e., market power, preference, and cost parameters):

$$P_{h}=\frac{\lambda \;\theta_{a}\theta_{h}+c_{a}\theta_{h}+\left(1+\theta_{a}\right)c_{h}}{1+\theta_{h}+\theta_{a}}$$ (19)

$$P_{a}=\frac{\lambda \;\theta_{a}\left(1+\theta_{h}\right)+\theta_{a}c_{h}+\left(1+\theta_{h}\right)c_{a}}{1+\theta_{h}+\theta_{a}}$$ (20)

Substituting *P*_*h*_ and *P*_*a*_ in Eqs (15) and (16), we get the equilibrium quantities/market shares as:

$$X_{h}=\frac{\lambda \;\theta_{a}+c_{a}-c_{h}}{\lambda \left(1+\theta_{h}+\theta_{a}\right)}$$ (21)

$$X_{a}=\frac{\lambda \;\left(1+\theta_{h}\right)+c_{h}-c_{a}}{\lambda \;\left(1+\theta_{h}+\theta_{a}\right)}$$ (22)

Finally, using the equilibrium prices and quantities, we can derive the food suppliers’ profits as:

$$\pi_{h}=(P_{h}-c_{h})X_{h}=\frac{\left[\lambda \theta_{a}\theta_{h}+c_{a}\theta_{h}-c_{h}\theta_{h}\right]\left[\lambda \theta_{a}+c_{a}-c_{h}\right]}{\lambda {\left(1+\theta_{h}+\theta_{a}\right)}^{2}}$$ (23)

$$\pi_{a}=(P_{a}-c_{a})X_{a}=\frac{\left[\lambda \theta_{a}\left(1+\theta_{h}\right)+c_{h}\theta_{a}-c_{a}\theta_{a}\right]\left[\lambda \left(1+\theta_{h}\right)+c_{h}-c_{a}\right]}{\lambda {\left(1+\theta_{h}+\theta_{a}\right)}^{2}}$$ (24)

#### 2.1.3. Comparative statics

Eqs (11) and (12) show the interdependence between the FAH and the FAFH options as the price of an option is a direct argument in the demand for its substitute. Additionally, an increase in the suppliers’ cost and/or market power increases the prices of both dining options (i.e., $\frac{\partial P_{h}}{\partial c_{a}}>0,\;\frac{\partial P_{h}}{\partial c_{h}}>0,0\frac{\partial P_{h}}{\partial \theta_{a}}>0,\;\frac{\partial P_{h}}{\partial \theta_{h}}>0$ and $\frac{\partial P_{a}}{\partial c_{h}}>0,\;\frac{\partial P_{a}}{\partial c_{a}}>0,\;\frac{\partial P_{a}}{\partial \theta_{h}}>0,\;\frac{\partial P_{a}}{\partial \theta_{a}}>0$), while the greater the market power and/or the costs in a market, the lower is the equilibrium quantity/market share of this dining option and the higher is the equilibrium quantity/market share of its substitute (i.e., $\frac{\partial x_{h}}{\partial c_{h}}\langle 0,\;\;\frac{\partial x_{h}}{\partial c_{a}}\rangle 0$ and $\frac{\partial x_{a}}{\partial c_{a}}\langle 0,\;\frac{\partial x_{a}}{\partial c_{h}}\rangle 0$ and $\;\frac{\partial x_{a}}{\partial \theta_{a}}\langle 0,\;\;\frac{\partial x_{a}}{\partial \theta_{h}}\rangle 0$ and $\frac{\partial x_{h}}{\partial \theta_{h}}\langle 0,\;\frac{\partial x_{h}}{\partial \theta_{a}}\rangle 0$).

### 2.2. Post-portion size reduction (post-PSR)

This section considers the case where the PSR policy is introduced in the market for FAFH. Without being provided with additional information regarding the reason behind this intervention, it is expected that consumers will reduce their valuation of food prepared away from home, which will result in a lower utility associated with FAFH. Also, by reducing the portion size of FAFH, suppliers of this product are expected to face lower costs. Following the same approach developed in the benchmark case, we begin by analyzing the behavior of consumers and food suppliers and deriving the market equilibrium conditions after PSR introduction. The market and welfare effects of PSR can, then, be determined by comparing the equilibrium conditions to those before the introduction of PSR.

#### 2.2.1. Consumer utility

The consumer utility function after the introduction of PSR is:

$$\begin{array}{cc} U_{a}=U-P_{a}+\lambda^{\prime }\alpha & \text{if}\;\text{a}\;\text{reduced}\;\text{portion}\;\text{meal}\;\text{prepared}\;\text{away}\;\text{from}\;\text{home}\;\text{is}\;\text{consumed}\\ U_{h}=U-P_{h} & \text{if}\;\text{a}\;\text{meal}\;\text{prepared}\;\text{at}\;\text{home}\;\text{is}\;\text{consumed} \end{array}$$ (25)

where *λ*′ is the reduced utility enhancement factor associated with the consumption of FAFH after the reduction of its portion size. In this context, the *utility/demand effect* of PSR is given by $\gamma =\lambda -\lambda^{\prime }$ where *λ*′ ≤ *λ*. The smaller is the portion size, the lower is the consumers’ valuation of FAFH, the smaller is *λ*′, and the greater is the utility effect of PSR *γ*. All other variables are as defined previously.

#### 2.2.2. Supplier costs

Due to PSR, suppliers of FAFH face lower costs, $c_{a}^{'}$. This *cost/supply effect* of PSR is given by $\rho =c_{a}-c_{a}^{'}$ where $c_{a}^{'}<c_{a}$. The smaller is the portion size, the smaller is $c_{a}^{'}$, and the greater is *ρ*. The cost faced by grocery stores/suppliers of ingredients for FAH, *c*_*h*_, is unaffected by the policy.

#### 2.2.3. Market equilibria

Substituting $c_{a}^{'}$ and *λ*′ for *c*_*a*_ and *λ* in the previous equilibrium conditions, we get the equilibrium prices, quantities/market shares and suppliers’ profits after the introduction of the PSR policy as:

$$P_{h}^{'}=\frac{\lambda '\theta_{h}+c_{a}^{'}\theta_{h}+\left(1+\theta_{a}\right)\;c_{h}}{1+\theta_{h}+\theta_{a}}$$ (26)

$$P_{a}^{'}=\frac{\lambda '\theta_{a}\left(1+\theta_{h}\right)+\theta_{a}c_{h}+\left(1+\theta_{h}\right)c_{a}^{'}}{1+\theta_{h}+\theta_{a}}$$ (27)

$$X_{h}^{'}=\frac{\lambda '\;\theta_{a}+c_{a}^{'}-c_{h}}{\lambda '\left(1+\theta_{h}+\theta_{a}\right)}$$ (28)

$$X_{a}^{'}=\frac{\lambda '\left(1+\theta_{h}\right)+c_{h}-c_{a}^{'}}{\lambda '\left(1+\theta_{h}+\theta_{a}\right)}$$ (29)

$$\pi_{h}^{'}=\left(P_{h}^{'}-c_{h}\right)X{'}_{h}=\frac{\left[\lambda '\theta_{a}\theta_{h}+c_{a}^{'}\theta_{h}-c_{h}\theta_{h}\right]\left[\lambda '\theta_{a}+c_{a}^{'}-c_{h}\right]}{\lambda {\left(1+\theta_{h}+\theta_{a}\right)}^{2}}$$ (30)

$$\pi_{a}^{'}=\left(P_{a}^{'}-c_{a}^{'}\right)X_{'}^{a}=\frac{\left[\lambda '\theta_{a}\left(1+\theta_{h}\right)+c_{h}\theta_{a}-c_{a}^{'}\theta_{a}\right]\left[\lambda '\left(1+\theta_{h}\right)+c_{h}-c_{a}^{'}\right]}{\lambda '{\left(1+\theta_{h}+\theta_{a}\right)}^{2}}$$ (31)

### 2.3. Market and welfare effects of PSR

In this section we analyze the market and welfare effects of reduced portion size of FAFH under different scenarios that are likely to emerge with the introduction of the policy.

#### 2.3.1. Market effects of PSR

The reduced suppliers’ cost and consumer valuation that follow the introduction of the portion size reduction for FAFH cause the equilibrium price of FAFH to fall, i.e., $P_{a}^{'}<P_{a}$.

Since a cost reduction (cost effect) results in increased output, while a decrease in consumer valuation (utility effect) decreases the equilibrium quantity of FAFH, the effect of PSR on the quantity/market share of FAFH depends on the relative magnitude of the cost and utility effects. In particular, a relatively high (low) cost effect and/or a relatively low (high) utility effect of PSR will result in increased (reduced) quantity of the FAFH. Comparing the equilibrium quantities of FAFH before and after PSR (i.e., Eqs (22) and (29)), shows that when the cost effect of PSR, *ρ*, is greater (lower) than the threshold value given by:

$$\rho^{*}=\gamma \frac{\left(c_{a}-c_{h}\right)}{\lambda }$$ (32)

the equilibrium quantity/market share of the FAFH increases (decreases) after the policy introduction (i.e., $X_{a}^{'}$⋛ *X*_*a*_ if *ρ* ⋛ ρ*). Note that the threshold value ρ* is a function of the utility effect. The greater is the utility effect, *γ*, the greater is ρ*, and the smaller is the likelihood that *ρ* > ρ* (and $X_{a}^{'}>X_{a}$).

Given that this is a covered market, any gains in the quantity/market share of FAFH due to PSR is equal to the losses in quantity/market share of FAH. Thus, the threshold value under which *X*_*a*_ increases is the same as the threshold value under which *X*_*h*_ decreases. Mathematically, comparing the equilibrium quantities of FAH before and after PSR (i.e., Eqs (21) and (28)), confirms that when the cost effect *ρ* is lower (greater) than the threshold value given by ρ* in Eq (32), the equilibrium quantity/market share of FAH will increase (decrease) after the PSR introduction, (i.e., $X_{h}^{'}⋛X_{h}$ if *ρ* ⋛ ρ*). The greater is the utility effect, *γ*, the greater is ρ*, and the greater the likelihood that ρ* > *ρ* (and $X_{h}^{'}>X_{h}$).

Similarly, comparing Eqs (19) and (26), we can derive the conditions under which the price of FAH will decrease (increase) post PSR. When the cost effect is greater than the threshold value given by:

$$\rho^{**}=\frac{-\gamma \theta_{a}\theta_{h}}{\theta_{h}}=-\gamma \theta_{a}$$ (33)

the equilibrium price of FAH will decrease. Given that *ρ* is positive, the inequality *ρ* > ρ** always holds, which means that, similar to *P*_*a*_, PSR causes *P*_*h*_ to always decrease. One could hypothesize that when PSR causes the demand for FAFH to decrease, the demand for FAH will increase and, given that the cost for grocery stores is unchanged, the price of FAH should increase in this situation. However, as Eq (17) indicates, *P*_*h*_ is a function of *P*_*a*_ only (and not *λ*). *P*_*h*_ is positively related to *P*_*a*_, which implies that, post PSR, the reduction in *P*_*a*_ will cause *P*_*h*_ to also decrease. *P*_*h*_ becomes a function of *λ* only when we substitute *P*_*a*_ into it (see Eq (19)), which implies that *P*_*h*_ is affected by *λ* only through the effect of *λ* on *P*_*a*_. At equilibrium, *P*_*h*_ is positively related to *λ* and *c*_*a*_ and, given that both parameters fall because of PSR, so does *P*_*h*_. Graphically, the reduction in *P*_*a*_ reduces the intercept of the demand for FAH in Eq (11), while the reduced *λ* reduces the (absolute value of the) slope of this inverse demand function *D*_*h*_. The outcome is a counterclockwise rotation of *D*_*h*_, which, for given costs *c*_*h*_, results in reduced *P*_*h*_ (on this, see also discussion on Fig 5 below).

**Fig 3:**
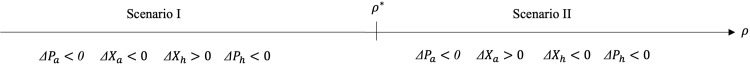
Market effects of the introduction of PSR for FAFH.

Consequently, post PSR we have two possible scenarios. While always decreasing *P*_*a*_ and *P*_*h*_, the introduction of PSR can: i) decrease *X*_*a*_ and increase *X*_*h*_ (Scenario I when *ρ* < ρ*); and ii) increase *X*_*a*_ and decrease *X*_*h*_ (Scenario II when *ρ* > ρ*). A schematic representation of the two scenarios depicting the possible market effects of the introduction of PSR is shown in Fig 3 below. In Scenario I the utility effect dominates the cost effect, while in Scenario II the cost effect becomes the dominant one.

Graphically, the introduction of PSR for FAFH causes a downward parallel shift of the marginal cost curve, *c*_*a*_ (cost effect), and reduces the intercept and the absolute value of the slope of the demand for FAFH (utility effect). As noted earlier, the reduced price and consumer valuation of the FAFH under PSR cause a decrease in the intercept and in the absolute value of the slope of the demand for FAH.

The market effects of PSR can also be presented in the consumer utility space. In this setting, the reduced *P*_*a*_ and *λ* increase the intercept and reduce the slope of the *U*_*a*_ utility curve, while a reduced *P*_*h*_ causes an upward parallel shift of the *U*_*h*_ utility curve.

**Fig 4:**
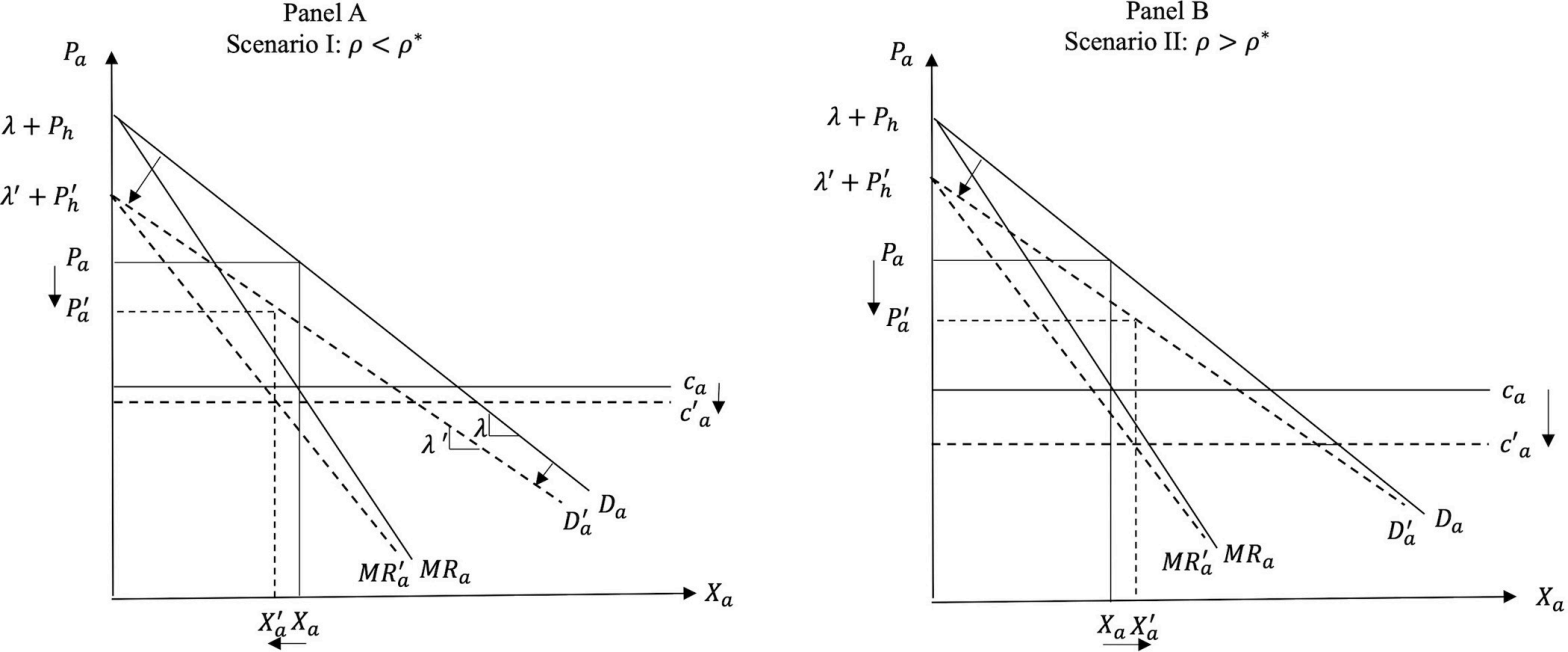
Effects of PSR on FAFH.

**Fig 5:**
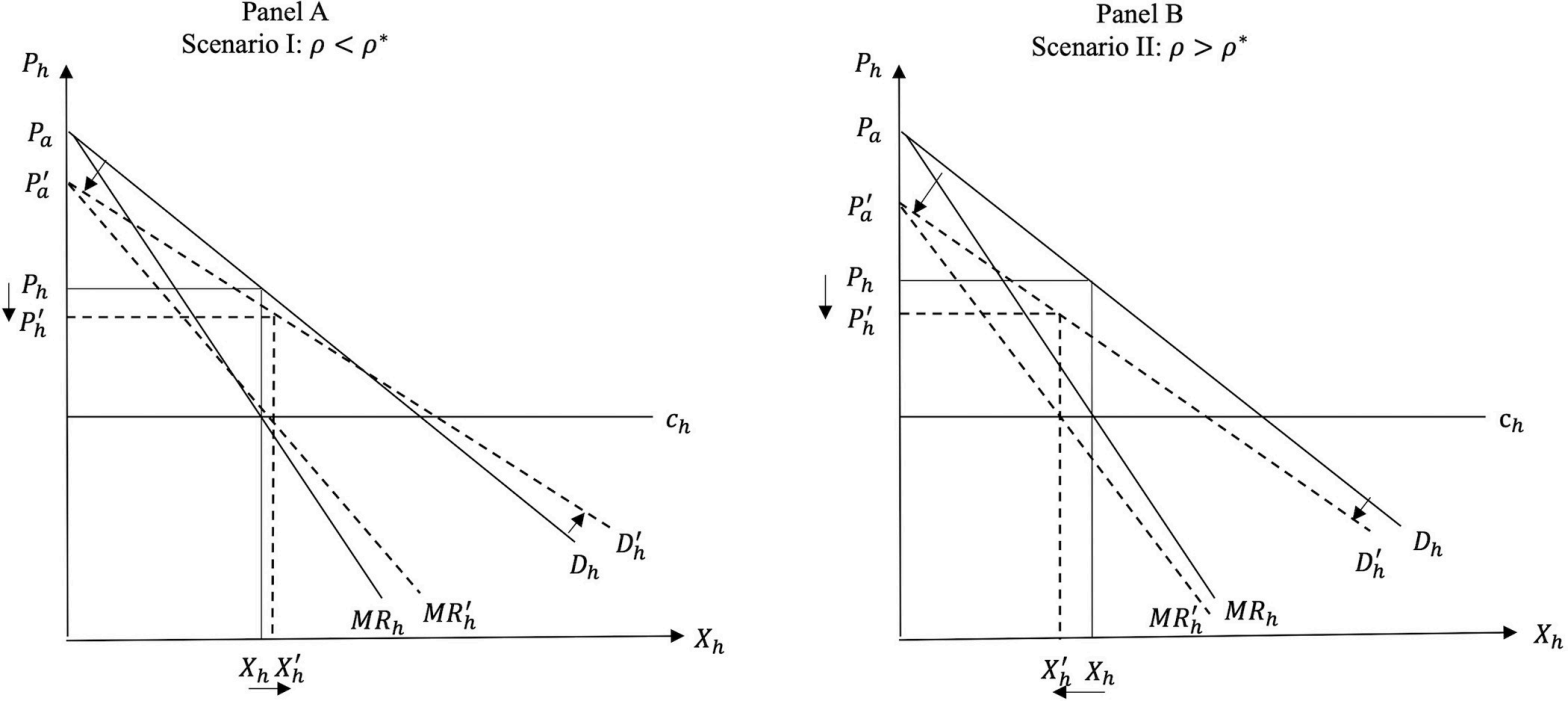
Effects of PSR on FAH.

**Fig 6:**
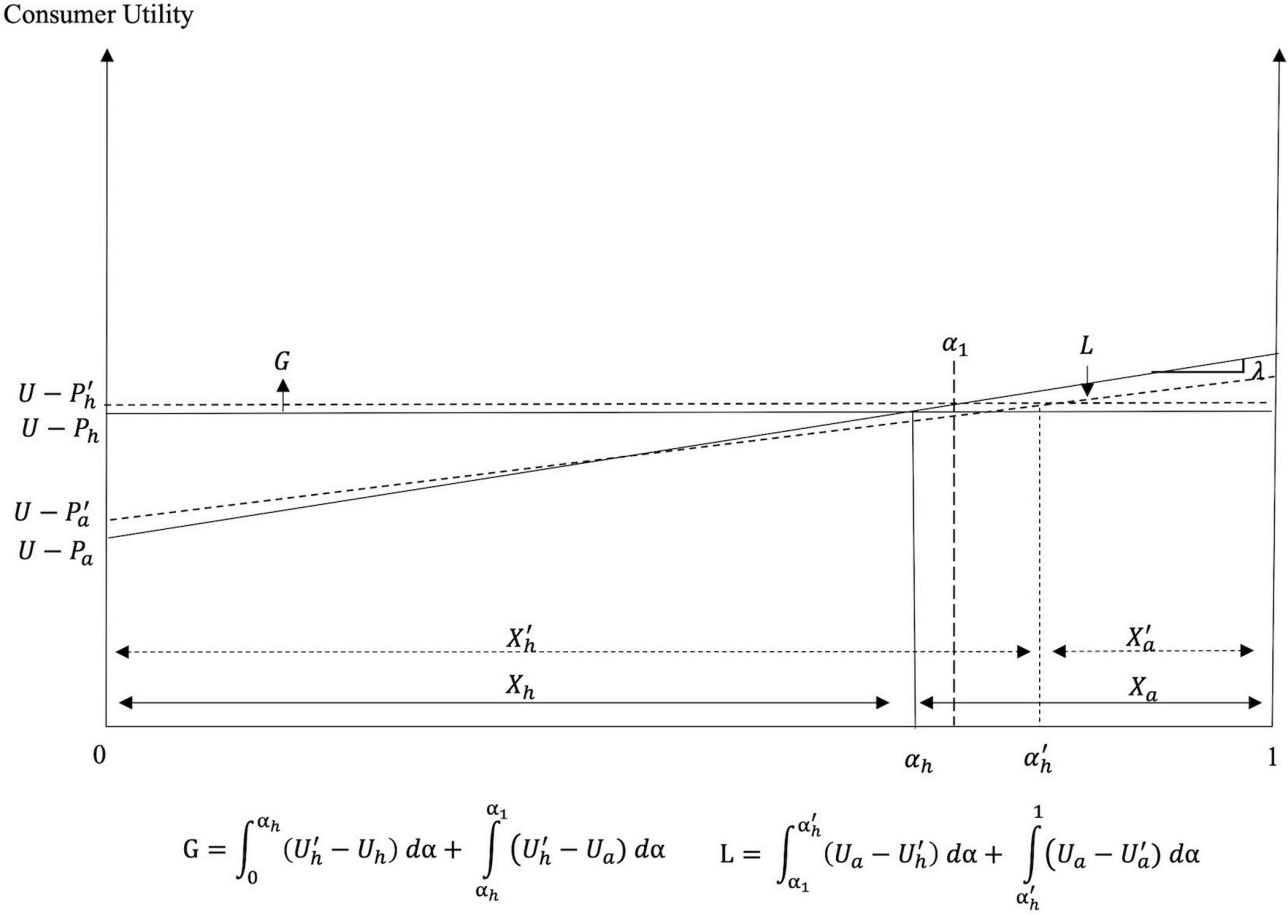
Consumption decisions and welfare under PSR in Scenario I (*ρ* < ρ*).

**Fig 7:**
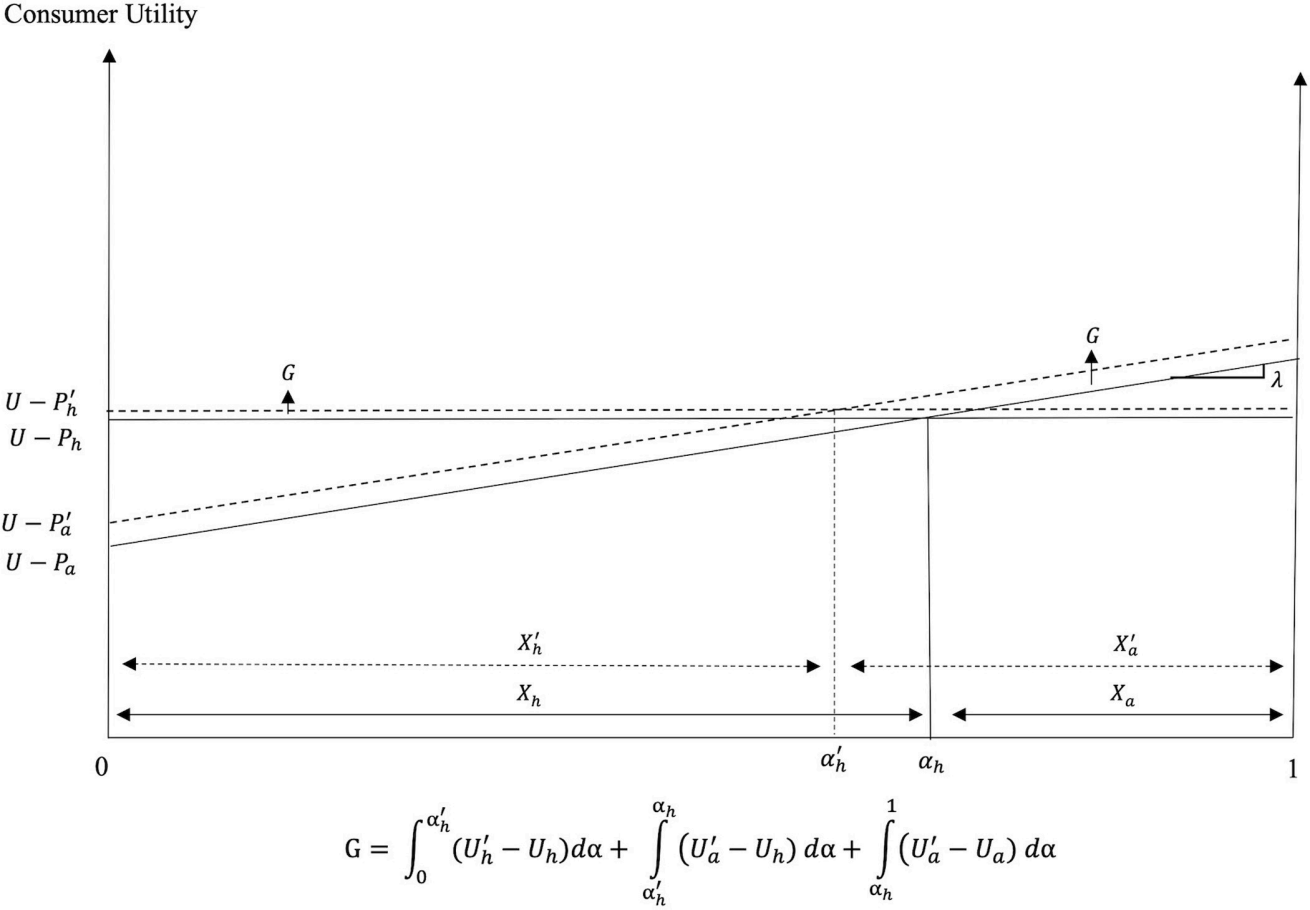
Consumption decisions and welfare under PSR in Scenario II (*ρ* > ρ*).

Figs 4 and 5 show the market effects of PSR on FAFH and FAH, respectively, under the two scenarios in the price-quantity space, while Figs 6 and 7 depict Scenarios I and II in the consumer utility space. The solid and dashed lines in these figures depict the relevant demand, cost, and utility schedules before and after PSR, respectively.

In panel A of Fig 4, the decrease in quantity *X*_*a*_ means that the utility effect of PSR (i.e., the reduced consumer valuation of FAFH) outweighs the cost effect, while in panel B, where the cost effect outweighs the utility effect, the equilibrium quantity of FAFH increases post PSR policy.

As noted earlier, due to the interdependence between the two dining options, the decrease in the consumer valuation and cost of the FAFH due to PSR policy decrease the intercept and the absolute value of the slope of the demand for FAH (*D*_*h*_). Panel A of Fig 5 graphs Scenario I, while panel B graphs Scenario II, where the introduction of PSR in the market for FAFH causes both the quantity (*X*_*h*_) and the price (*P*_*h*_) of FAH to decrease.

The market effects of the introduction of PSR for FAFH can be summarized as follows:

**Result 1:** The introduction of PSR for FAFH decreases the prices of both FAFH and FAH.

**Result 2:** The impact of PSR on the quantities/market shares of FAFH and FAH depends on the relative magnitude of the cost and utility effects of PSR. The greater (smaller) the cost reduction of FAFH and/or the smaller (greater) the decrease in the consumer valuation of food prepared away from home under PSR, the greater the likelihood that the policy introduction will increase (decrease) the equilibrium quantity/market share of FAFH and will decrease (increase) the equilibrium quantity/market share of FAH.

#### 2.3.2. Welfare effects of PSR on consumers and food suppliers

The effects of the PSR policy on consumer welfare under the two scenarios are illustrated in Figs 6 and 7. Regions marked as *G* and *L* depict the consumer welfare gains and losses, respectively, from the policy introduction. The mathematical expressions for the welfare gains and losses associated with each scenario are also included in these figures.

The introduction of PSR creates winners and losers among consumers. In Scenario I, where the utility effect outweighs the cost effect, all consumers who consume FAFH before and after the policy introduction (i.e., consumers with *α* ∈ (*α*_1_, 1]) experience welfare losses. Consumers with relatively stronger preference for FAFH (those with higher *α* values) realize greater welfare losses compared to consumers with moderate preference for dining out. Consumers with relatively weak preference for FAFH (i.e., consumers with *α* ∈ (*α*_h_, *α*_1_)) who decide to switch from FAFH to FAH and those who consume FAH before and after PSR, gain after the policy introduction. On the other hand, when the cost effect outweighs the utility effect, as in Scenario II, all consumers gain. The greater the cost effect and/or the smaller the utility effect of PSR, the greater the decrease in *P*_*a*_ and *P*_*h*_, and the greater the consumer welfare gains from the policy. Although all consumers gain in Scenario II, the welfare gains differ across consumers as consumers with relatively weaker preference for FAFH (those with lower *α* values) enjoy greater welfare gains from PSR than consumers with relatively stronger preference for this dining option (i.e., those with higher *α* values).

The food suppliers’ gains and losses in each scenario in the FAH and the FAFH markets are also illustrated in Figs 4 and 5. Mathematically, comparing the profits of food service suppliers before and after PSR (i.e., Eqs (24) and (31), respectively) shows that the effect of introducing the PSR policy for FAFH on supplier’s profits depends on the relative magnitude of the utility and cost effects of PSR, the strength of the consumer preference for dining out, suppliers’ market power, and the initial costs in both markets, *c*_*a*_ and *c*_*h*_. *Ceteris paribus*, the greater the cost effect and/or the lower the utility effect of PSR, the greater the likelihood that *X*_*a*_ will increase and this increase will outweigh the negative impact of *P*_*a*_ reduction. When the cost effect is greater (less) than the threshold value given by:

$$\rho_{1}=\frac{-\frac{2\theta_{h}\left[\left(\lambda -\gamma \right)\left(1+\theta_{h}\right)+c_{h}-c_{a}\right]}{\left(\lambda -\gamma \right){\left(1+\theta_{h}+\theta_{a}\right)}^{2}}+\sqrt{{\left(\frac{2\theta_{a}\left[\left(\lambda -\gamma \right)\left(1+\theta_{h}\right)+c_{h}-c_{a}\right]}{\left(\lambda -\gamma \right){\left(1+\theta_{h}+\theta_{a}\right)}^{2}}\right)}^{2}-4\left\{\frac{\theta_{a}}{\left(\lambda -\gamma \right){\left(1+\theta_{h}+\theta_{a}\right)}^{2}}\right\}\left\{\frac{\;\gamma \;\theta_{a}\left(\;{\left(\;c_{h}-c_{a}\right)}^{2}-\lambda \;\left(\lambda -\;\gamma \right){\left(1+\theta_{h}\right)}^{2}\right)}{\lambda \;\left(\lambda -\gamma \right){\left(1+\theta_{h}+\theta_{a}\right)}^{2}}\right\}}}{2\frac{\theta_{a}}{\left(\lambda -\gamma \right){\left(1+\theta_{h}+\theta_{a}\right)}^{2}}}$$ (34)

the profits of FAFH suppliers increase (decrease) with the introduction of PSR, i.e., *π’*_*a*_ ⋛ *π*_*a*_ if *ρ* ⋛ *ρ*_1_.

Similarly, comparing Eqs (23) and (30) that correspond to the profits of the FAH suppliers before and after PSR, respectively, shows that when the cost effect is greater (less) than the threshold value given by:

$$\rho_{2}=\frac{-\frac{-2\;\theta_{a}\left(\left(\lambda -\gamma \right)\;\theta_{a}+c_{a}-c_{h}\right)}{\left(\lambda -\gamma \right){\left(1+\theta_{h}+\theta_{a}\right)}^{2}}-\sqrt{{\left(\frac{-2\;\theta_{a}\left(\left(\lambda -\gamma \right)\;\theta_{a}+c_{a}-c_{h}\right)}{\left(\lambda -\gamma \right)\;{\left(1+\theta_{h}+\theta_{a}\right)}^{2}}\right)}^{2}-4\left\{\frac{\theta_{h}}{\left(\lambda -\gamma \right)\;{\left(1+\theta_{h}+\theta_{a}\right)}^{2}}\right\}\left\{\frac{\gamma \;\theta_{h}\left(\;{\left(\;c_{a}-c_{h}\right)}^{2}-\lambda \;\left(\lambda -\gamma \right)\theta_{a}{}^{2}\right)}{\lambda \;\left(\lambda -\gamma \right)\;{\left(1+\theta_{h}+\theta_{a}\right)}^{2}}\right\}}}{2\frac{\;\theta_{h}}{\left(\lambda -\;\gamma \right)\;{\left(1+\theta_{h}+\theta_{a}\right)}^{2}}}$$ (35)

the profits of FAH suppliers decrease (increase) with the introduction of PSR, i.e., *π’*_*h*_ ⋛ *π*_*h*_ if *ρ* ⋛ *ρ*_2_.

The reasoning is as follows. Our findings indicate that the introduction of PSR for FAFH causes *P*_*a*_ and *P*_*h*_ to always decrease. In addition, regarding the equilibrium quantities in the two markets, the smaller the cost effect (*ρ*) and the higher the utility effect (*γ*), the greater the likelihood that *X*_*h*_ will increase and *X*_*a*_ will decrease after PSR. Given the decreased *P*_*a*_, the higher the likelihood that *X*_*a*_ will decrease, the higher the likelihood that the profits of FAFH suppliers will also decrease. Thus, FAFH suppliers always lose under Scenario I (panel A in Fig 4 where *X*_*a*_ decreases post PSR), while the suppliers of FAH always lose under Scenario II (panel B in Fig 4) where the cost effect is relatively stronger than the utility effect and *X*_*h*_ falls due to PSR.

The welfare impacts of PSR can be summarized as follows:

**Result 3:** Consumers of FAH always realize welfare gains after the introduction of PSR policy for FAFH due to the reduced price of this dining option.

**Result 4:** The effect of PSR on consumers of FAFH depends on their valuation of this dining option and its price, *P*_*a*_. The greater (lower) the cost effect and the lower (greater) the utility effect of PSR, the greater (lower) the likelihood that consumers will benefit from the policy. The lower the consumers’ valuation of food prepared away from home, the greater their benefits from the policy.

**Result 5:** The effect of PSR policy for FAFH on the suppliers of FAH and FAFH depends on the relative magnitude of the cost and utility effects of PSR, and suppliers’ costs and market power. The smaller (greater) the cost effect and/or the greater (smaller) the utility effect, the higher the likelihood that PSR introduction will result in losses for the suppliers of FAFH (FAH).

## 3. Impacts of information provision on incidence of PSR

The welfare gains of PSR identified in the previous section can be considered as the minimum gains from the policy as consumers do not internalize the extra benefits (e.g., health and environmental benefits) associated with reduced portion sizes. However, as noted in the introduction of this paper, previous studies have shown that large portion sizes lead to increased food intake and obesity, which is associated with an elevated risk of several major non-communicable diseases (NCDs), including type 2 diabetes, heart disease, stroke, asthma, and several cancers. In addition to providing health benefits, previous studies also show that reducing the plate size reduces food waste (which is an important source of greenhouse gas emissions that cause climate change) by about 20%.

In this section, we are trying to capture these extra benefits of the PSR policy by accounting for possible consumer responses to information provision about links between portion size and obesity, food waste, and climate change. The provision of this information can be expected to affect consumers’ response to reduced portion sizes. In essence, in this section we are assuming that information will make consumers endogenize, at least some of, the health and environmental benefits associated with the reduced portion size of FAFH.

In this context, two cases can emerge: Case 1 where the consumer valuation of FAFH in the presence of information, *λ*”, is greater than or equal to the consumer valuation under no information but still lower that the valuation prior to the policy, i.e., the consumer valuation of FAFH falls due to the PSR but less than it would fall in the absence of information, *λ’* < *λ”* < *λ*; and Case 2, where the provision of information on the impact of portion size on obesity, food waste and climate change, increases the consumer valuation of the resized FAFH relative to the benchmark case, i.e., *λ”* > *λ*.

The rest of this section focuses on the impact of information provision on the market and welfare effects of PSR in these two cases.

### 3.1. Case 1 (*λ’* < *λ”* < *λ*)

Assuming that information has a positive impact on consumer valuation of the resized FAFH products (i.e., *λ”* > *λ*′), the utility effect *γ’* = *λ* – *λ”* is weaker than that in the absence of information (*λ’* < *λ”* < *λ*). Given the continued presence of the cost effect of PSR faced by FAFH suppliers, the equilibrium prices of FAFH and FAH will always decrease (i.e., *P”a* < *P*_*a*_ and *P”*_*h*_ < *p*_*h*_), while the impact of PSR on the equilibrium quantities of the two dining options will depend on the relative magnitude of the utility and the cost effects of PSR. In other words, the results are qualitatively similar to those in the no information case with the weakening of the utility effect making more likely the emergence of Scenario II.

**Result 6:** When information provision reduces the utility effect of PSR, it increases the likelihood of the emergence of a scenario characterized by a relatively dominant cost effect of the policy. The greater the impact of information on the utility effect, the more likely the emergence of Scenario II (where PSR increases *X*_*a*_ and decreases *X*_*h*_), the greater the likelihood that all consumers of FAH and FAFH experience welfare gains, and that information provision results in gains (losses) for the suppliers of FAFH (FAH).

### 3.2. Case 2 (*λ”* > *λ*)

In this case, information provision increases the consumer valuation of food prepared away from home, *λ”*, which reverses the negative impact of the utility effect, i.e., *γ*” = *λ*”– *λ* > 0.

#### 3.2.1. Market and welfare impacts of PSR

An increase in the consumer valuation of the FAFH, *λ*, increases the utility associated with the consumption of this product (see Eq (1)) and attracts previous consumers of food prepared at home to the market for FAFH. At the same time, the cost effect of PSR reduces the cost faced by FAFH suppliers causing the quantity/market share of FAFH, *X*_*a*_, to always increase in this case. As this is a covered market, the increase in *X*_*a*_ results in the quantity of FAH, *X*_*h*_, to always decrease in this case. On the other hand, the impact of the policy on prices, *P*_*a*_ and *P*_*h*_, depends on the relative magnitude of the cost and utility effects of PSR. In particular, since the cost effect of PSR results in reduced *P*_*a*_ while the utility effect of the policy causes *P*_*a*_ to increase, the higher the cost effect and/or the lower the utility effect of PSR, the greater is the likelihood that the policy will result in reduced *P*_*a*_. As *P*_*h*_ is positively related to *P*_*a*_, a reduction in *P*_*a*_ will cause *P*_*h*_ to also decrease and vice versa (see below).

**Fig 8:**
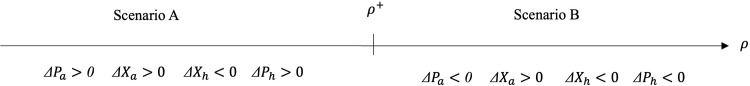
Market effects of the introduction of PSR with information provision in Case 2.

Substituting $c_{a}^{'}$ and *λ”* for *c*_*a*_ and *λ* in Eqs (19)—(24) we get the equilibrium prices, quantities/market shares and the suppliers’ profits in Case 2. Comparing the equilibrium prices of the FAFH before and after the policy in Case 2 shows that, when the cost effect of PSR, *ρ*, is lower (greater) than the threshold value given by:

$$\rho^{+}=\gamma^{\prime \prime }\theta_{a}$$ (36)

the equilibrium price of FAFH increases (decreases) after the policy introduction (i.e., *P”*_*a*_ ⋛ *P*_*a*_ if *ρ* ⋛ *ρ*^+^). The greater the increase in the consumer valuation of food prepared away from home in Case 2, the greater the utility effect, *γ”*, the greater is *ρ*^+^, and the greater the likelihood that *ρ* < *ρ*^+^ (and $P_{a}^{''}>P_{a}$). Similarly, comparing the equilibrium prices of FAH before and after the policy in Case 2, shows that when the cost effect of PSR, *ρ*, is lower (greater) than the threshold value *ρ*^+^ (Eq (36)), the equilibrium price of FAH increases (decreases) after the policy introduction (i.e., *P”*_*h*_ ⋛ *P*_*h*_ if *ρ* ⋛ *ρ*^+^). Consequently, post-PSR post-information we have two possible scenarios depicted in Fig 8.

**Fig 9:**
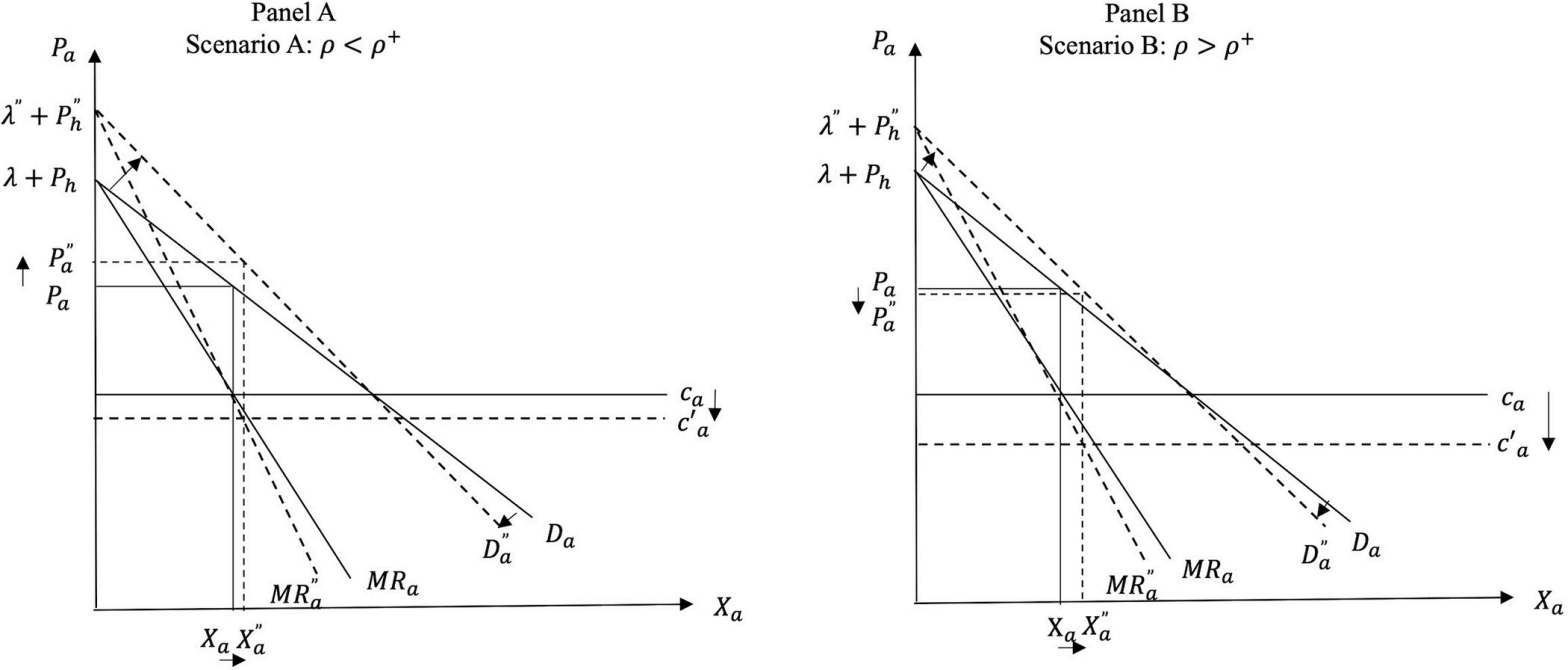
Effects of PSR with information provision on FAFH in Case 2.

**Fig 10:**
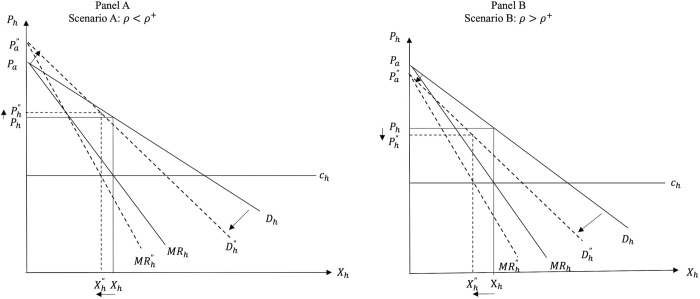
Effects of PSR with information provision on FAH in Case 2.

**Fig 11:**
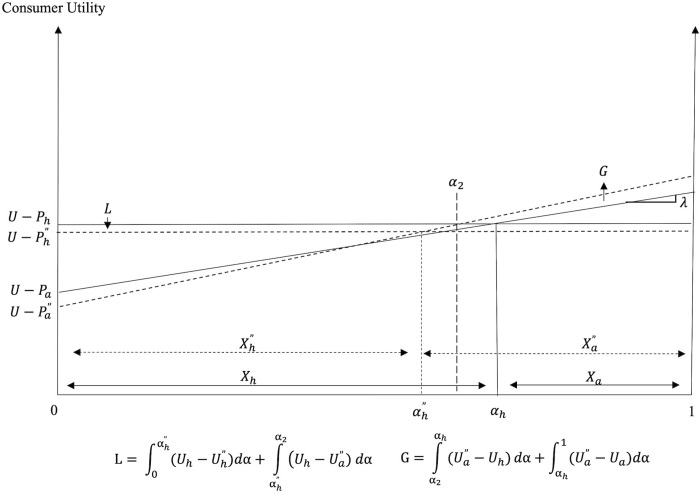
Consumption decisions and welfare in Scenario A of Case 2 (*ρ* < *ρ*^+^).

**Fig 12:**
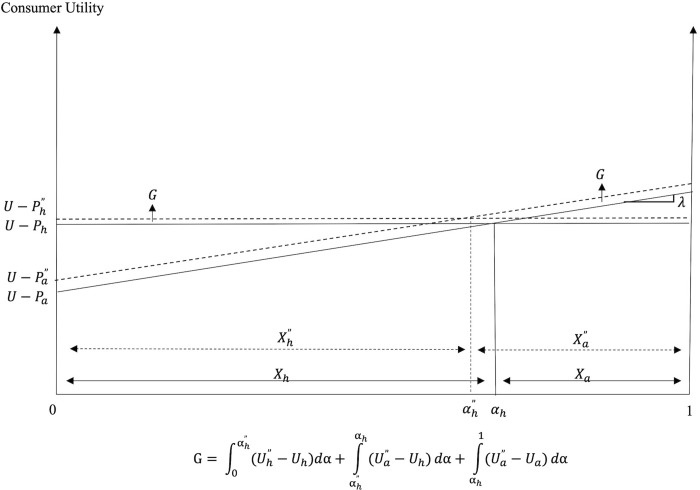
Consumption decisions and welfare in Scenario B of Case 2 (*ρ* > *ρ*^+^).

Graphically, Figs 9 and 10 show the market effects of PSR on FAFH and FAH under the two scenarios in Case 2, while Figs 11 and 12 depict Scenarios A and B, respectively, in the consumer utility space.

The market effects of PSR with information provision in Case 2 can be summarized as follows:

**Result 7:** When the provision of information about the health and environmental benefits of PSR increases the consumer valuation of FAFH, the quantity/market share of FAFH increases while the quantity/market share of the FAH decreases.

**Result 8:** When the provision of information about the health and environmental benefits of PSR increases the consumer valuation of FAFH, the effect of PSR on the prices of FAH and FAFH depends on the relative magnitude of the cost and utility effects. The greater (smaller) the increase in the utility associated with the consumption of FAFH and/or the smaller (greater) the cost effect of PSR in Case 2, the greater the likelihood that the policy will increase (decrease) the equilibrium prices of FAFH and FAH.

Regarding the impact of the policy on consumer welfare, when PSR with information provision cause *P*_*a*_ and *P*_*h*_ to increase (Scenario A in Fig 11), consumers who choose to eat at home before and after PSR (i.e., consumers with $\alpha \in \left[0,\alpha_{h}^{''}\right]$), and those who switch from FAH to FAFH and have a relatively weak preference for food prepared away from home (i.e., consumers with $\alpha \in \left(\alpha_{h}^{''},\alpha_{2}\right]$) lose. Consumers who switch from home prepared meals to food prepared away from home and have intermediate preference for FAFH (i.e., consumers with *α* ∈ (*α*_*2*_, *α*_*h*_]), realize welfare gains in Case 2 and so do consumers who dine away from home before and after the policy (i.e., consumers with *α* ∈ (*α*_*h*_, 1]). On the other hand, when PSR with information provision cause *P*_*a*_ and *P*_*h*_ to decrease (Scenario B in Fig 12), all consumers realize welfare gains. The greater the cost effect relative to the utility effect, the greater the decrease in *P*_*a*_ and *P*_*h*_, and the greater the consumer welfare gains.

The impact of PSR with information provision on the food suppliers is illustrated in Figs 9 and 10. FAFH suppliers always gain due to the increased demand and the reduced costs in the FAFH market. In particular, in Scenario A (panel A of Fig 9), FAFH suppliers always gain as both *P*_*a*_ and *X*_*a*_ increase. In Scenario B (panel B of Fig 9), despite the decrease in *P*_*a*_, FAFH suppliers also gain as the increased demand for FAFH makes up for the decrease in profit margins due to the reduced price. On the other hand, FAH suppliers always lose. In Scenario A (panel A of Fig 10), FAH suppliers lose as the decrease in *X*_*h*_ outweighs the increase in *P*_*h*_, while in Scenario B (panel B of Fig 10), FAH suppliers lose as both *P*_*h*_ and *X*_*h*_ decrease.

The welfare effects of PSR with information provision in Case 2 on consumers and food suppliers can be summarized as follows:

**Result 9:** Consumers with relatively strong preference for food prepared away from home realize welfare gains under PSR when information provision increases the consumer valuation of this dining option.

**Result 10:** The effect of PSR with information provision on consumers who prefer food prepared at home before and after the policy and those who switch from FAH to FAFH depends on the effect of this policy on the prices of these dining options. In particular, the greater (lower) the cost effect and the lower (greater) the utility effect, the greater the likelihood that the PSR policy will decrease (increase) the prices of FAH and FAFH and will result in welfare gains (losses) for these groups of consumers.

**Result 11:** PSR with information provision causes FAFH suppliers to always gain and FAH suppliers to always lose under the policy.

Before concluding this section, it is important to note that, as pointed out by an anonymous reviewer, the provision of information about the links between portion size and food intake, obesity, food waste and climate change could also affect the portion size of food prepared at home. Even though among the reasons many consumers prefer FAH is their ability to control the quantity (and quality) of the food they consume, it is possible that the provision of the information about the benefits of reduced portion sizes would result in, at least some of them, reducing the portion size of the food they prepare at home. It turns out that the implications of a FAH portion size reduction as a result of information provision are straightforward and can be examined in the context of the economic model developed and utilized for our analysis.

In particular, a reduction of the portion size of FAH would reduce the demand faced by the grocery stores/suppliers of ingredients for FAH, *D*_*h*_, which would reduce, in turn, the price of FAH, *P*_*h*_, and the profits of FAH suppliers, *Π*_*h*_. The reduced price *P*_*h*_ would increase the utility associated with the consumption of FAH, *U*_*h*_, the welfare of consumers that prefer food prepared at home, *CW*_*h*_, and would attract to FAH previous consumers of FAFH. The reduction in the share of consumers preferring FAFH, *X*_*a*_, would reduce, in turn, the demand faced by the suppliers of FAFH, *D*_*a*_, and, with it, the price of FAFH, *P*_*a*_, and the profits of suppliers of FAFH, *Π*_*a*_. The reduced *P*_*a*_ would increase, then, the utility associated with the consumption of FAFH, *U*_*a*_, and the welfare of consumers that prefer food prepared away from home, *CW*_*a*_, and would limit the number of consumers that find it optimal to switch to food prepared at home. Overall, a reduction in the portion size of FAH as the result of information provision would reduce the prices of the two dining options, increase the share of consumers preferring food prepared at home, benefit consumers, and hurt the food product suppliers. Equally important, these results hold no matter the impact of information provision on the consumer valuation of FAFH, i.e., no matter whether information provision results in Case 1 or Case 2 examined earlier.

## 4. Simulation analysis

The objective of the simulation analysis is to quantify the system-wide market and welfare effects of PSR derived earlier. Using the consumer demands for FAH and FAFH pre-PSR (given by Eqs (3) and (4), respectively) and observed data on prices, expenditure shares and profit margins, we estimate the exogenous parameters of our model (*λ*, *c*_*h*_, *c*_*a*,_ *θ*_*h*_ and *θ*_*a*_). Once the model is calibrated, we determine the impact of PSR introduction in the FAFH market under different values of (i) the cost of FAFH (*c*_*a*_) and (ii) the consumer valuation for FAFH (*λ*).

### 4.1. Data

Despite the overall increase in consumer spending on food between 1997 and 2019, spending on FAH increased at a slower rate (39.7%) compared to FAFH (60.5%) [23]. While available, we do not use 2020 data as the Covid-19 pandemic had an unprecedented impact on the supply chain and the demand patterns in the food system [24]. For instance, many restaurants had limited capacity or closed entirely due to Covid-19 and food delivery apps became a prominent source of food prepared away from home during the pandemic [25–29]. As USDA data considers mail ordered and delivery food as FAH, this data was not included in our simulation analysis as the PSR policy considered in our study would apply to all food prepared away from home, including the delivered food.

The consumer price of a home prepared meal (*P*_*h*_) is based on the USDA monthly cost of FAH for a moderate plan for an adult and averages at around $3 in 2019 [30]. The moderate plan is one of four meal plans designed by the USDA to help Americans shop smarter during the Great Depression and provides a diet consistent with that of most people. On the other hand, the consumer price of a meal consumed away from home (*P*_*a*_) is based on the USDA quarterly FAFH prices (not including taxes) for a moderate plan in 2012 (the most recent year available) [31]. Accounting for an average inflation rate of 1.6% per year between 2012 and 2019 [32], and a 5.52% average sales tax rate in 2019 [33], results in a moderate meal away from home costing around $13.

The quantities/market shares of FAH (*X*_*h*_) and FAFH (*X*_*a*_) are estimated using the USDA food expenditure series data. The USDA provides yearly data, from 1997 through 2020, on FAH expenditures for all sellers (e.g., grocery stores, convenience stores, warehouse clubs and supercenters, direct selling by farmers, etc.) and FAFH expenditures for all sellers (e.g., full-service restaurants, limited-service restaurants, hotels and motels, recreational places, etc.). The expenditure shares (ES) for FAH and FAFH are 45% and 55%, respectively, in 2019 [34]. Using the formulas/relationships $ES_{h}=\frac{P_{h}\;X_{h}}{P_{h}\;X_{h}+P_{a}\;X_{a}},\;X_{a}=1-X_{h}$ and the FAH and FAFH meal prices discussed above, we estimate the market shares of FAH (*X*_*h*_) and FAFH (*X*_*a*_) at 73% and 27%, respectively.

The supplier costs of a meal of FAH (*c*_*h*_) and a meal of FAFH (*c*_*a*_) are estimated using data on the grocery stores and food services profit margins. In the US, grocery stores operate at very low profit margins. Most grocery store chains/companies have hundreds of locations, which enable them to operate at scale and make money by selling large quantities. According to Cohan, the average grocer’s net profit margin increased slightly from 1.4% in 2014 to 1.7% in 2019 [35]. Profit margins of fast-food restaurants (6%-9%), full services restaurants (2%-6%), food trucks (6%-9%), caterings and events (7%-8%) are collected from different sources [36–39] and averaged to reflect a profit margin in the FAFH industry of 6%. The historically slim profit margin in the restaurants industry is a chronic challenge that stems from a highly competitive market environment and a consumer price sensitivity [40].

The data used for the model calibration and simulation are presented in Table 1 below.

**Table 1:** Data and calibrated parameters.

Benchmark Parameters	Value
*Data*:	
*P*_*h*_: price of a meal of FAH	$3
*P*_*a*_: price of a meal of FAFH	$13
*X*_*h*_: market share/quantity of FAH	0.73 (73%)
*X*_*a*_: market share/quantity of FAFH	0.27 (27%)
*Calibrated Exogenous Parameters*:	
*λ*: utility enhancement factor associated with the consumption of FAFH	13.7
*c*_*h*_: marginal cost of FAH per meal	$2.95
*c*_*a*_: marginal cost of FAFH per meal	$12.22
*θ*_*h*_: market power of FAH suppliers	0.005
*θ*_*a*_: market power of FAFH suppliers	0.2

### 4.2. Simulation results

Our analysis assumes that PSR for FAFH results in reduced consumer valuation (*λ*) of this dining option and lower costs faced by FAFH suppliers (*c*_*a*_). However, once consumers are provided with information on the policy’s rationale (i.e., health and environmental benefits), the analysis assumes that two cases can emerge. Case 1, where consumer valuation of FAFH falls due to the PSR but less than it would fall in the absence of information. Results in this Case are qualitatively similar to those in the absence of information with an increased likelihood for the emergence of a scenario characterized by a relatively weak utility effect/dominant cost effect (Scenario II). In Case 2, the feeling of supporting a good cause and one’s health increases *λ* relative to the benchmark case changing the market and welfare effects of PSR.

The simulation results show that for all possible decreases in the values of *λ* and *c*_*a*_, post-PSR both *P*_*h*_ and *P*_*a*_ always decrease, with the percentage decrease in *P*_*a*_ exceeding that of *P*_*h*_ as *P*_*h*_ is affected by *λ* only through the effect of *λ* on *P*_*a*_ (recall Eqs (13) and (14)). For market shares/quantities, any decrease (increase) in *X*_*a*_ is coupled with the same increase (decrease) in *X*_*h*_. For instance, if the consumer valuation decreases by 20% due to PSR, the cost effect threshold value that determines the condition under which different scenarios emerge is ρ* ≈ 17%. For all cost effect values, *ρ*, that are greater (smaller) than ρ*, the equilibrium quantity/market share of FAFH increases (decreases) and the market share of FAH decreases (increases). Simulation results also reveal that when *λ* decreases by 20%, FAFH suppliers make gains (Δ*π*_*a*_ > 0) when *ρ* > *ρ*_1_ ≈ 16%, and FAH suppliers gain (Δ*π*_*h*_ > 0) when *ρ* < *ρ*_2_ ≈ 7%.

Table 2 summarizes the percentage changes in prices, quantities, supplier profits and consumer welfare under different scenarios. For illustration purposes, in Scenario I, *λ* decreases by 20% and *c*_*a*_ decreases by 5% (i.e., the utility effect dominates the cost effect); in Scenario II, *λ* decreases by 5% and *c*_*a*_ decreases by 20% (i.e., the cost effect outweighs the utility effect); while in Scenario A of Case 2 *λ* increases by 20% and *c*_*a*_ decreases by 5%; and in Scenario B of Case 2 *λ* increases by 5% and *c*_*a*_ decreases by 20%. Case 1 under information provision is not considered separately as its results are identical to those of Scenario II.

**Table 2:** Market and welfare effect of PSR and information.

	*Post-PSR*		*Post-PSR*, *Post information*: *Case 2*	
	Scenario I Δ*λ* = -20% Δ*c*_*a*_ = -5%	Scenario II Δ*λ* = -5% Δ*c*_*a*_ = -20%	Scenario A Δ*λ* = +20% Δ*c*_*a*_ = -5%	Scenario B Δ*λ* = +5% Δ*c*_*a*_ = -20%
Price of a meal of FAH (Δ*P*_*h*_)	-0.2%	-0.35%	+0.05%	-0.3%
Price of a meal of FAFH (Δ*P*_*a*_)	-7.5%	-16.6%	+0.5%	-14.8%
Market share/quantity of FAH (Δ*X*_*h*_)	+13%	-17.4%	-17.1%	-23%
Market share/quantity of FAFH (Δ*X*_*a*_)	-34.5%	+46.3%	+45.7%	+61.5%
Profit of FAH suppliers (Δ*π*_*h*_)	+2%	-35.1%	-17.5%	-37.8%
Profit of FAFH suppliers (Δ*π*_*a*_)	-65.7%	+103.4%	+154.6%	+174%
Welfare of FAH consumers	+0.7%	+0.6%	-0.5%	+0.6%
Welfare of FAFH consumers switching to FAH	-22%	NA	NA	NA
Welfare of FAH consumers switching to FAFH	NA	+5.5%	+0.5%	+7.3%
Welfare of FAFH consumers of FAFH	-8.3%	+8.6%	+11.8%	+20.2%

All changes are expressed in percentages (%)

NA denotes non applicable

Note that, while both dining option prices decrease post PSR, the impact of the policy is significantly greater on the price of FAFH (compare the *P*_*a*_ reductions of 7.5% and 16.6% in Scenarios I and II, respectively, to *P*_*h*_ decreases of 0.2% and 0.35%). The profits of FAFH suppliers decrease by 65.7% in Scenario I, due to the reduced market share by 34.5% when the utility effect of PSR dominates the cost effect of the policy, and increase by 103.4% when the cost effect dominates and the market share of FAFH increases by 46.3%. While FAH consumer welfare is minimally affected by the policy (as their welfare gains do not exceed 0.7%), the welfare of FAFH consumers who switch to FAH under Scenario I decreases by 22%.

Consistent with our analytical results, information provision in Case 1 increases the likelihood for the emergence of Scenario II, while in Case 2, information provision significantly increases the demand for food prepared away from home by 45.7% and 61.5% in Scenarios A and B, respectively, which increases the relevant profits of FAFH suppliers by 154.6% and 174%. All consumers realize welfare gains in Case 2, with FAFH consumers benefiting the most from the policy as their welfare increases by 20.2% in Scenario B.

## 5. Summary and concluding remarks

This study develops models of heterogeneous consumer preferences for different dining options and imperfect competition among food suppliers to analyze the market and welfare effects of portion size reduction for food away from home. Different scenarios on the nature of differentiation between food at home and food away from home, the information available to consumers and their response to links between portion size and obesity, food waste, and climate change are considered within this framework.

The analysis considers the impacts of a reduction in portion size on both the demand and the supply sides of the market; i.e., the impact of PSR on the consumer valuation of FAFH (the products whose portion size is being reduced) (*utility effect*) and the costs faced by food service suppliers (*cost effect*).

The analysis shows that, while the reduced FAFH consumer valuation and supplier costs cause the prices of FAFH and FAH to always decrease after the introduction of PSR, the impact of the policy on the quantities/market shares of the FAFH and the FAH, consumer welfare and supplier profits is case-specific and dependent on the relative magnitude of the cost and utility effects of PSR, the strength of the consumer preference for dining out, and the food suppliers’ initial costs and degree of market power in the FAH and the FAFH markets. The greater (smaller) the cost effect and/or the smaller (greater) the utility effects of PSR, the greater the likelihood that the policy introduction will increase (decrease) the equilibrium quantity/market share of FAFH and will decrease (increase) the equilibrium quantity/market share of FAH.

Accounting for consumer heterogeneity in our model is essential for understanding the asymmetric welfare effects of PSR across consumers. Our study indicates that PSR creates winners and losers among customers. Generally, the lower the utility effect and/or the higher the cost effect of PSR, the higher the share of consumers who gain from the policy and the higher the consumer welfare gains. FAFH suppliers are shown to benefit from the policy when the impact of the reduced demand for FAFH is outweighed by the cost reduction faced by these suppliers. The lower (greater) the utility effect and/or the greater (lower) the cost effect, the higher is the gain for FAFH (FAH) suppliers.

The consumer welfare gains from PSR in the absence of information provision are the minimum gains from PSR as the extra benefits of the policy (i.e., benefits for consumers’ health and the environment) might not be internalized by consumers. However, previous studies have shown that large portion sizes lead to increased food intake/obesity, which is associated with an elevated risk of several major non-communicable diseases, including type 2 diabetes, heart disease, stroke, asthma, and several cancers. In addition to providing health benefits, previous studies have also shown that reducing the portion size reduces food waste (which is an important source of greenhouse emissions that cause climate change) by about 20%.

To capture the health and environmental benefits of PSR, the last part of the study assumes that, while facing the reduced portion size of FAFH, consumers are provided with information that links portion size with obesity, food waste, and climate change. In essence, this part of the study assumes that information will make consumers endogenize at least some of PSR’s extra benefits, which will increase consumer valuation of reduced portion sizes of food prepared away from home. The analysis shows that the results depend on the consumer responsiveness and reaction to this information.

In particular, if the consumer valuation of FAFH is greater than the consumer valuation in the absence of information but lower than the valuation prior to the policy, the results are qualitatively similar to those in the absence of information, with an increased likelihood for the emergence of a scenario characterized by a relatively weak utility effect/dominant cost effect. On the other hand, if the feeling of supporting a good cause and one’s health increases consumer valuation of the resized FAFH relative to the benchmark case, the results change. In this case, the quantity/market share of FAFH always increases, and the quantity/market share of FAH always decreases under PSR. The prices of FAFH and FAH increase when the increase in consumer valuation outweighs the decrease in costs and, in this case, only consumers with relatively strong preference for food prepared away from home realize welfare gains. On the other hand, when the prices decrease, all consumers realize welfare gains. Suppliers of FAFH are shown to always gain in this case, while the impact of the policy on FAH suppliers profits depends mainly on the relative magnitude of the cost and utility effects of PSR.

The results of our study were shown to be robust to the nature of the differentiation between the different dining options, while the market and welfare effects of the policy were quantified using a simulation analysis for all cases considered in our study.

In addition to providing insights on the market and welfare effects of PSR, our study provides policy makers with a systematic analysis that accounts for the key impacts of introducing PSR, such as changes in consumer valuation and costs, the value of information provision, and all possible scenarios and related outcomes that can emerge. With the results being dependent on the consumer reaction to PSR and the costs effects of the policy, our study can also provide a valuable theoretical grounding for empirical studies of certain portion size reductions in different economic environments. Finally, our study can provide the basis for the analysis of the economic causes and market and welfare consequences of portion size reduction when the latter is not mandated by a government policy but is, instead, a strategic choice of firms/food suppliers of food away from home.

## Supporting information

S1 Appendix FAH and FAFH are horizontally differentiated. (DOCX)
