# Supplementary material for: The Economics of a “portion size reduction” policy
Source: PLoS One. 2022 Dec 15;17(12):e0279165. doi: 10.1371/journal.pone.0279165 (PMC9754238; doi:10.1371/journal.pone.0279165)
Supplement: S1 Appendix — (DOCX) [file pone.0279165.s001.docx]

**Appendix**

**FAH and FAFH are horizontally differentiated**

The main analysis of this paper treats FAH and FAFH as vertically differentiated dining options, i.e., uniformly quality ranked by consumers so that, if offered at the same price, all consumers would prefer to have someone else, a professional, cook for them. This section considers the case in which the two dining options are horizontally differentiated, i.e., if offered at the same price, both dining options will enjoy positive market shares. Due to the market being covered (i.e., consumers will eat either at home or away from home), the results of our study are not affected by the nature of the differentiation between the two dining options.

In the case of horizontal differentiation, consumers are alike in their basic valuation (and willingness to pay) for the two dining options but, due to differences in tastes (i.e., some consumers enjoy cooking and/or want to control the quantity and quality of a meal while others prefer the restaurant experience) and/or locations (i.e., physical distance between homes and restaurants), consumers differ in their valuation of these two dining options. The consumer utility when FAH and FAFH are horizontally differentiated is given by:

$U_{a}=U-P_{a}+\lambda\alpha$ if a meal prepared away from home is consumed

$U_{h}=U-P_{h}+ \left( 1-\alpha\right)$ if a meal prepared at home is consumed (A1)

where the parameter is a non-negative utility enhancement factor associated with the consumption of FAH. All other variables are as defined previously. To ensure non-negative consumption shares for the two dining options, the preference parameters $\lambda$ and are such that $\lambda{>P}_{a}-P_{h}$ and ${>P}_{h}-P_{a}$.

Fig A1 graphs $U_{a}$ and $U_{h}$ and illustrates the decisions and welfare of consumers.


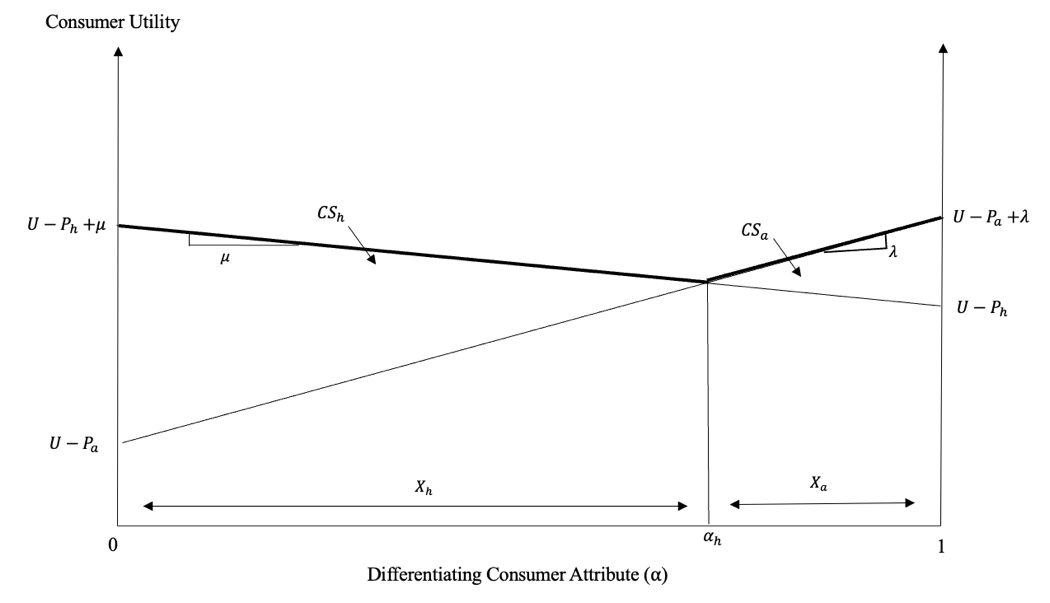


**Fig A1. Consumption decisions and welfare under pre-portion size reduction (PSR)**

The upward slopping curve graphs the utility levels when a meal prepared away from home is consumed, whereas the downward slopping curve graphs the utility when a meal prepared at home is consumed for different levels of the differentiating attribute $\alpha$ (i.e., for different consumers). Following the process developed in the case of vertical differentiation in the main part of this study, we can derive the market shares of, and consumer demands for FAH and FAFH, $X_{h}$ and $X_{a}$ in Fig A1, respectively, as:

$$X_{h}= \frac{P_{a}-P_{h}+}{\lambda+} (A2)$$

$$X_{a}= \frac{{\lambda-P}_{a}+P_{h}}{\lambda+} (A3)$$

As these demands are very similar to those in equations (3) and (4) (they are identical for $=0),$ the rest of the analysis and results are also the same with those in the case of vertical differentiation of FAH and FAFH. For completeness of exposition, equations (A4) - (A9) present the equilibrium prices, quantities, and supplier profits in the two markets per-PSR, while Figs A2-A9 depict the market and welfare impacts of PSR under the different cases considered in this study when FAH and FAFH are horizontally differentiated.

$$P_{h}=\frac{\theta_{a}\theta_{h}\lambda+\left( 1+\theta_{a} \right)\theta_{h}+\theta_{h}c_{a}+\left( 1+\theta_{a} \right)c_{h}}{1+\theta_{h}+\theta_{a}} (A4)$$

$$P_{a}=\frac{\theta_{a}\theta_{h}+\left( 1+\theta_{h} \right)\theta_{a}\lambda+\theta_{a}c_{h}+\left( 1+\theta_{h} \right)c_{a}}{1+\theta_{h}+\theta_{a}} (A5)$$

$$x_{h}=\frac{\left( 1+\theta_{a} \right)+\theta_{a}\lambda+c_{a}-c_{h}}{\left( 1+\theta_{h}+\theta_{a} \right)\left( \lambda+ \right)} (A6)$$

$$x_{a}=\frac{\left( 1+\theta_{h} \right)\lambda+\theta_{h}+c_{h}-c_{a}}{\left( 1+\theta_{h}+\theta_{a} \right)\left( \lambda+ \right)} (A7)$$

$$\pi_{h}=\frac{\left[ \theta_{a}\theta_{h}\lambda+\left( 1+\theta_{a} \right)\theta_{h}+\theta_{h}c_{a}-\theta_{h}c_{h} \right]\left[ \left( 1+\theta_{a} \right)+\theta_{a}\lambda+c_{a}-c_{h} \right]}{\left( \lambda+ \right)\left( 1+\theta_{h}+\theta_{a} \right)^{2}} (A8)$$

$$\pi_{a}=\frac{\left[ \theta_{a}\theta_{h}+\left( 1+\theta_{h} \right)\theta_{a}\lambda+\theta_{a}c_{h}-\theta_{a}c_{a} \right]\left[ \left( 1+\theta_{h} \right)\lambda+\theta_{h}+c_{h}-c_{a} \right]}{\left( \lambda+ \right)\left( 1+\theta_{h}+\theta_{a} \right)^{2}} (A9)$$

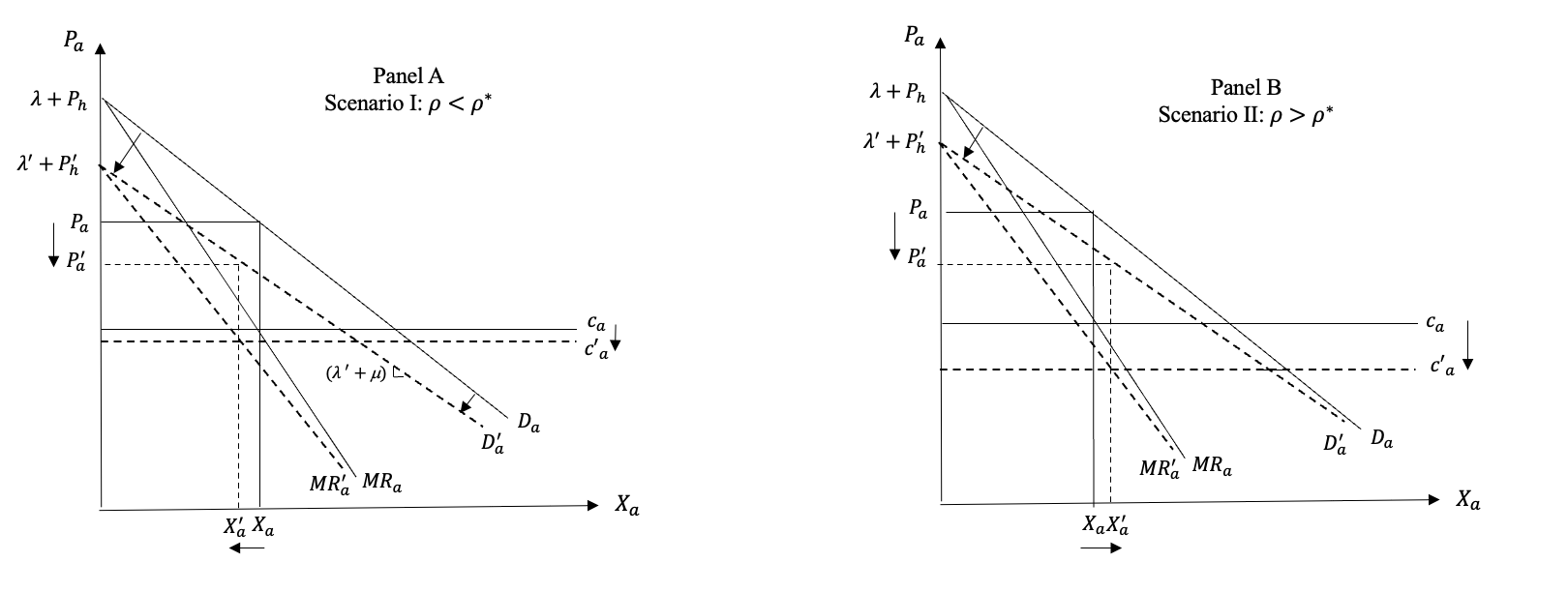


**Fig A2. Effects of PSR on FAFH**

**
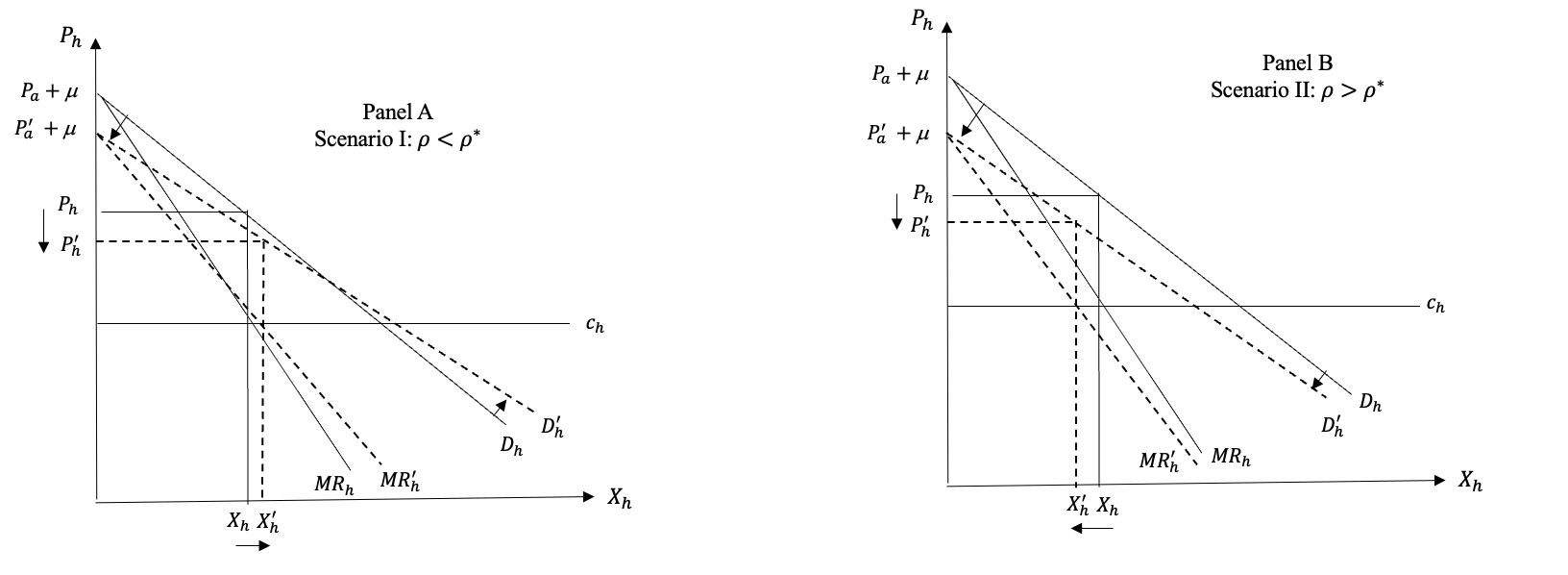
**

**Fig A3. Effects of PSR on FAH**

**
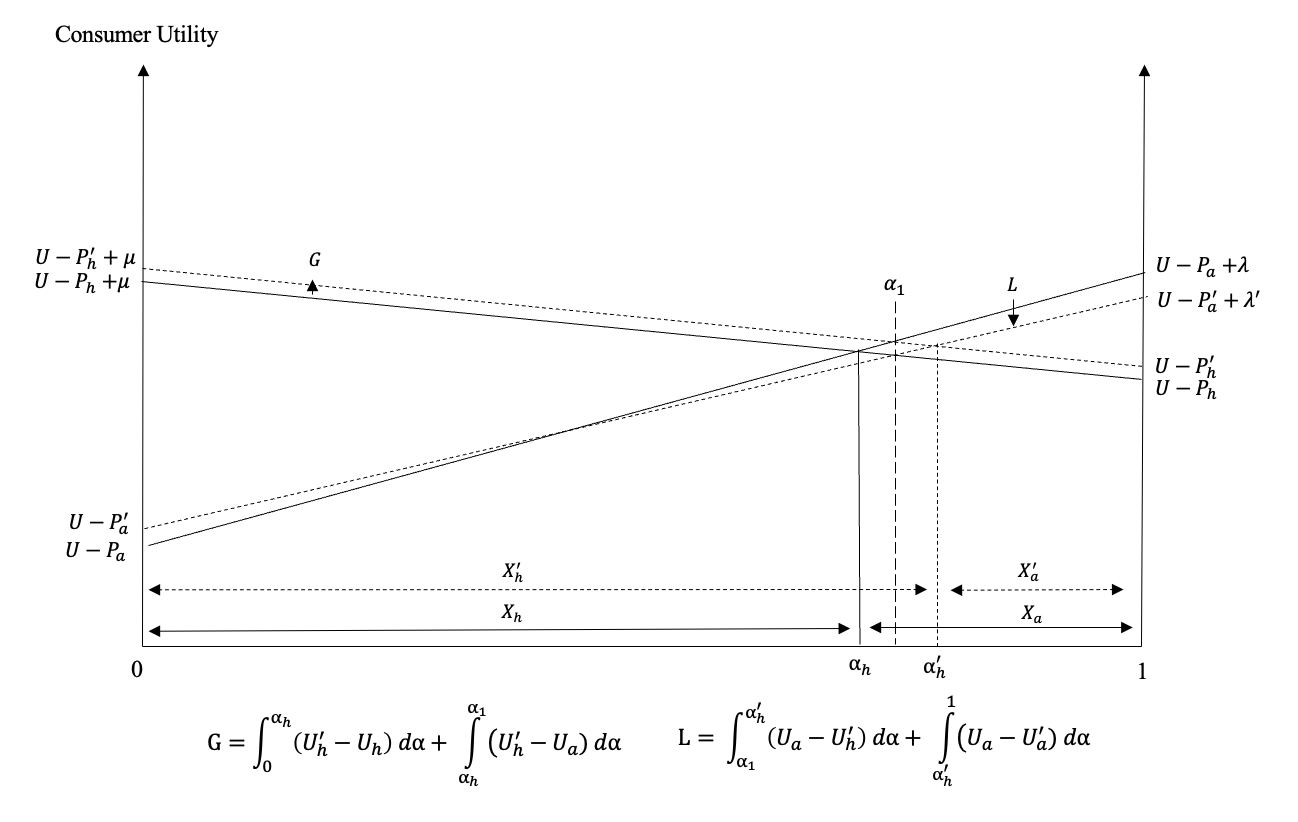
**

**Fig A4. Consumption decisions and welfare under PSR in Scenario I (**$\boldsymbol{\rho<}\boldsymbol{\rho}^{\boldsymbol{*}}$**)**

**
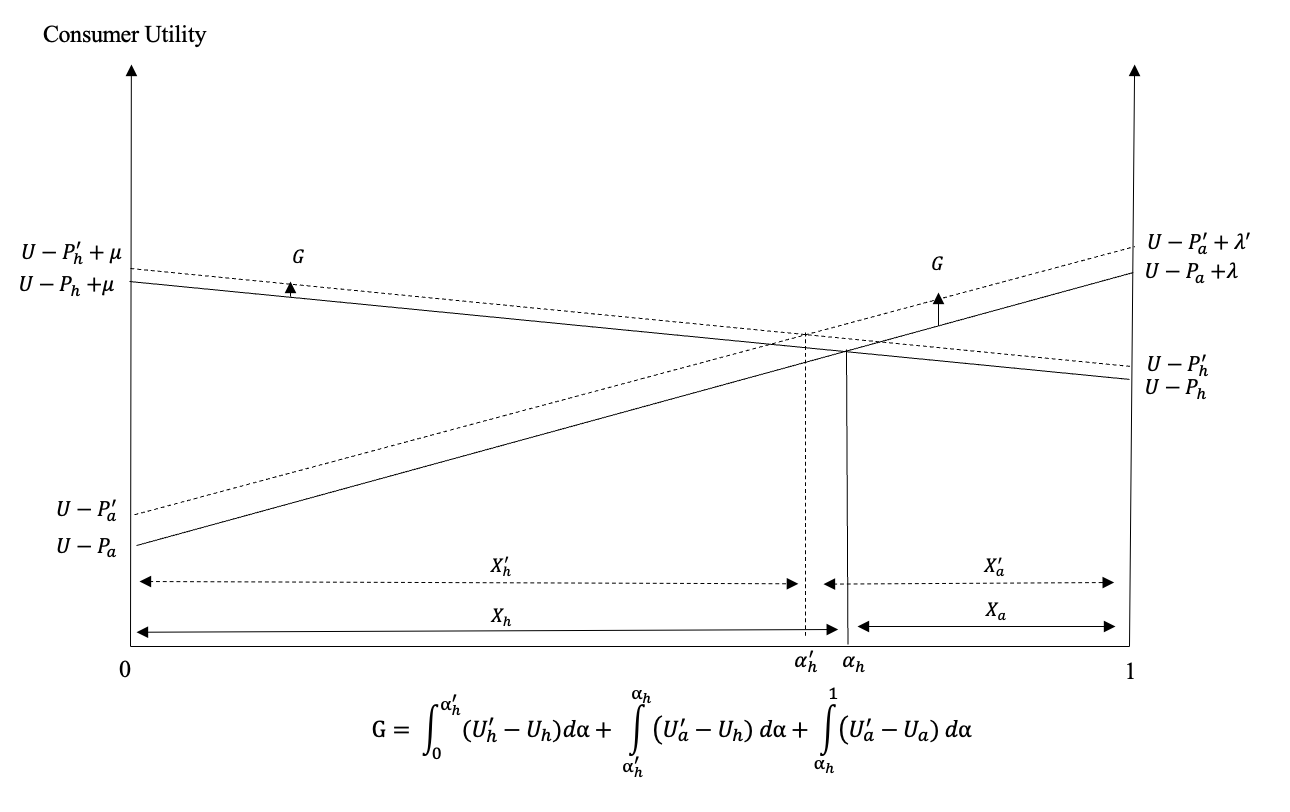
**

**Fig A5. Consumption decisions and welfare under PSR in Scenario II (**$\boldsymbol{\rho>}\boldsymbol{\rho}^{\boldsymbol{*}}$**)**

**
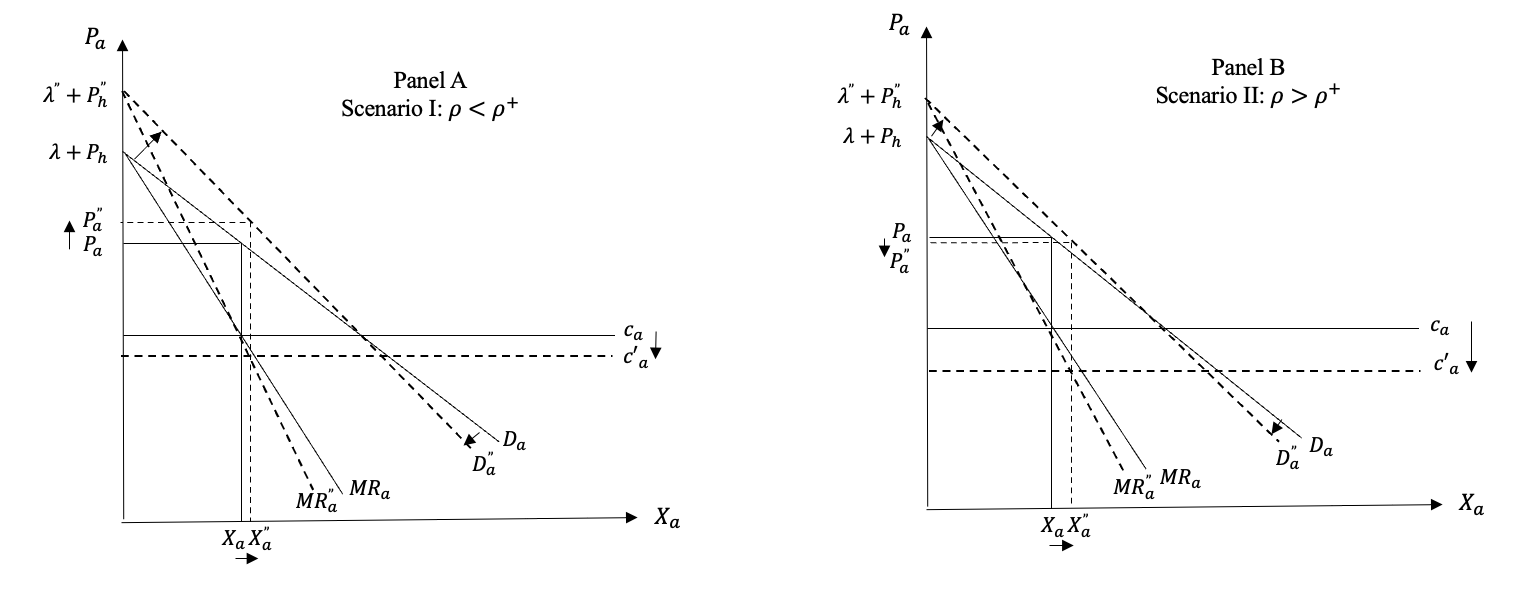
**

**Fig A6. Effects of PSR and information on FAFH in Case 2**

**
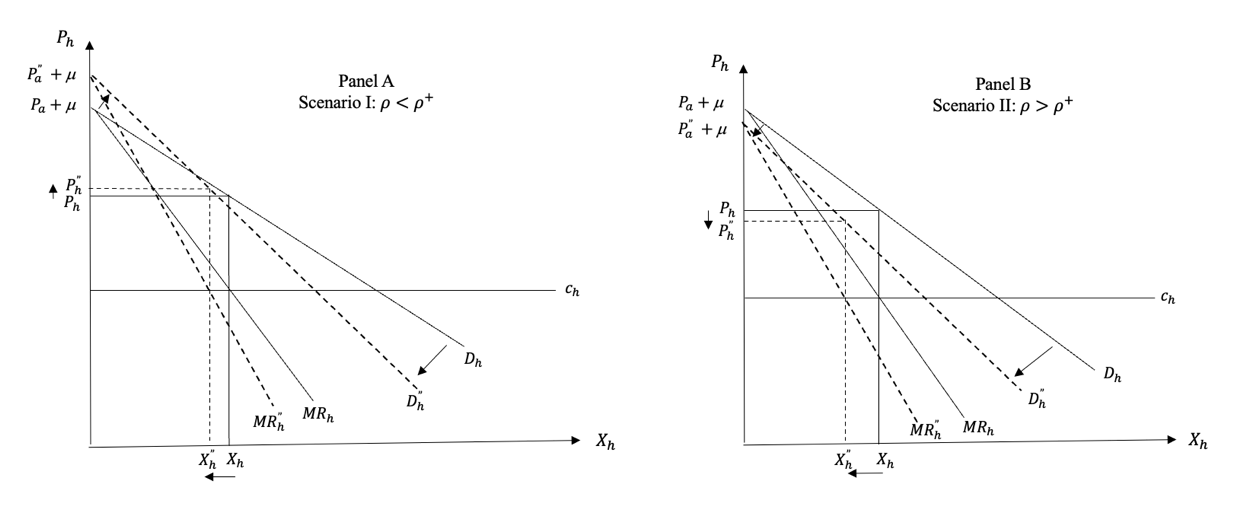
**

**Fig A7. Effects of PSR and information on FAH in Case 2**

**
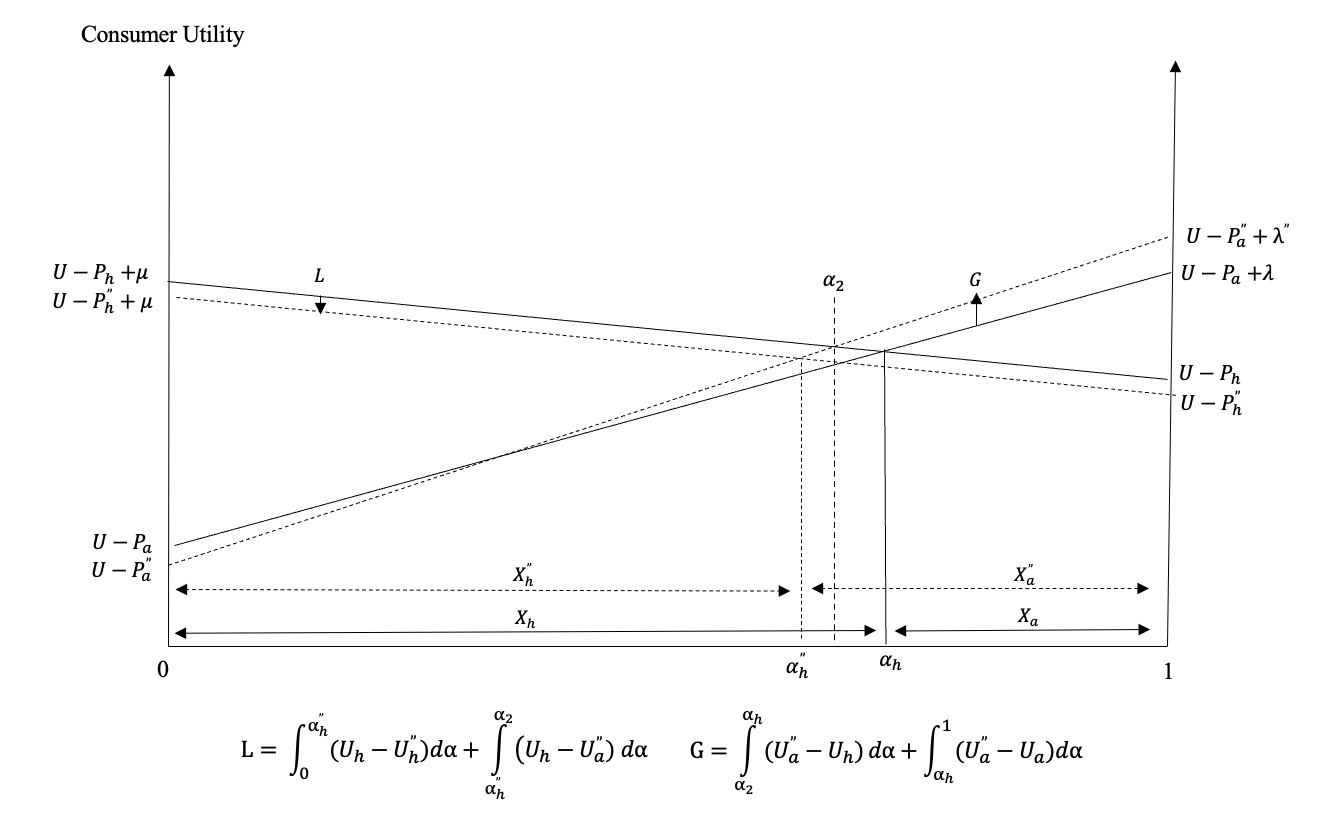
**

**Fig A8. Consumption decisions and welfare in Scenario A of Case 2 (**$\boldsymbol{\rho<}$ $\boldsymbol{\rho}^{\boldsymbol{+}}$**)**

**
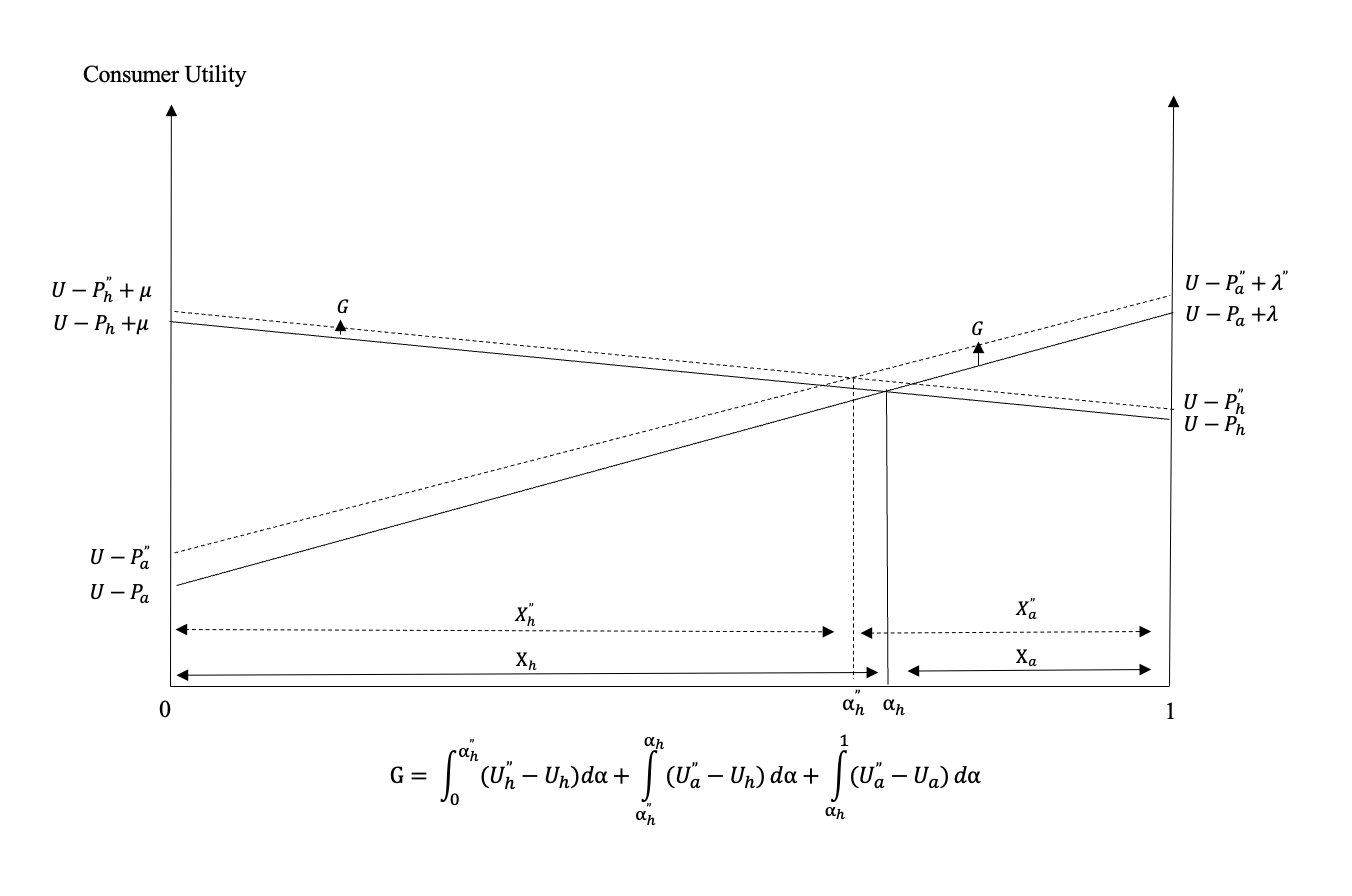
**

**Fig A9. Consumption decisions and welfare in Scenario B of Case 2 (**$\boldsymbol{\rho>}\boldsymbol{\rho}^{\boldsymbol{+}}$**)**
